# Supplementary figures and images for: The effect of sodium-glucose cotransporter 2 inhibitors in patients with chronic kidney disease with or without type 2 diabetes mellitus on cardiovascular and renal outcomes: A systematic review and meta-analysis
Source: PLoS One. 2023 Nov 29;18(11):e0295059. doi: 10.1371/journal.pone.0295059 (PMC10686459; doi:10.1371/journal.pone.0295059)

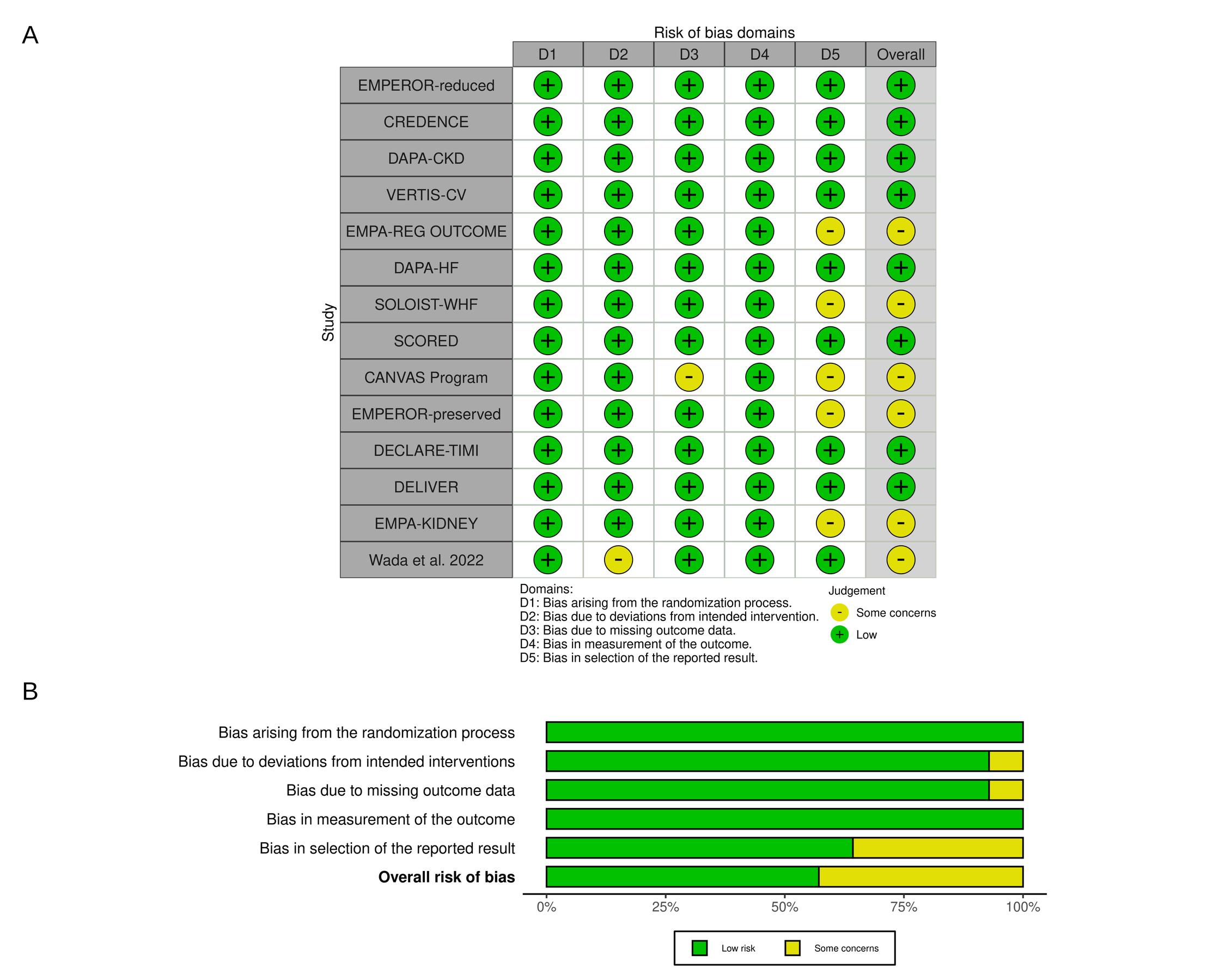

Supplement: S1 Fig — (A) Traffic light plot. (B) Bar plot. (TIF) [file pone.0295059.s002.tif]

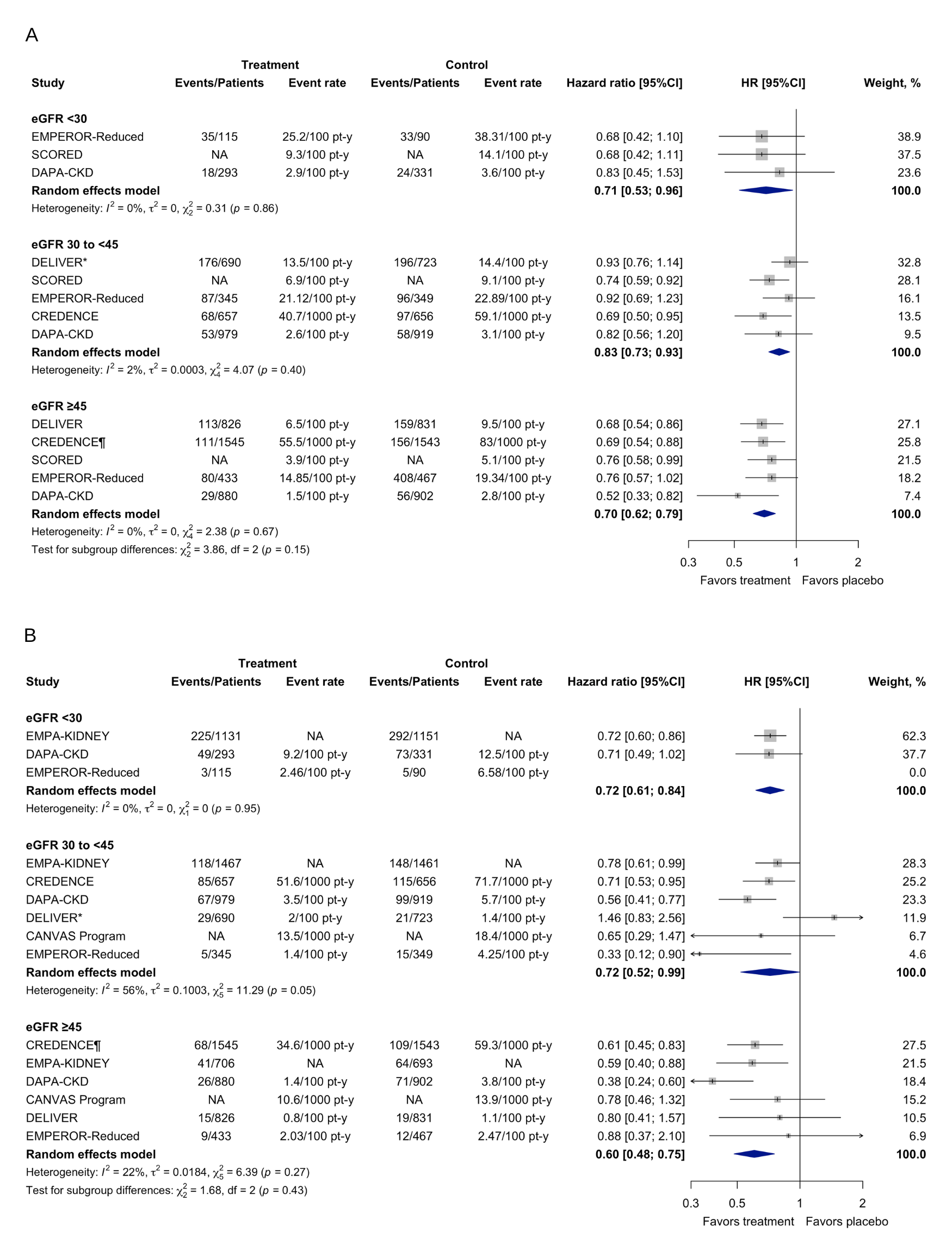

Supplement: S2 Fig — (A) Primary cardiovascular outcome. (B) Primary renal outcome. *Included patients with an eGFR of 25 to < 45 ml/min/1.73 m2. ¶Synthetic estimate created with a fixed effects model meta-analysis. CI, confidence interval; eGFR, estimated glomerular filtration rate; HR, hazard ratio; NA, not available. (TIF) [file pone.0295059.s003.tif]

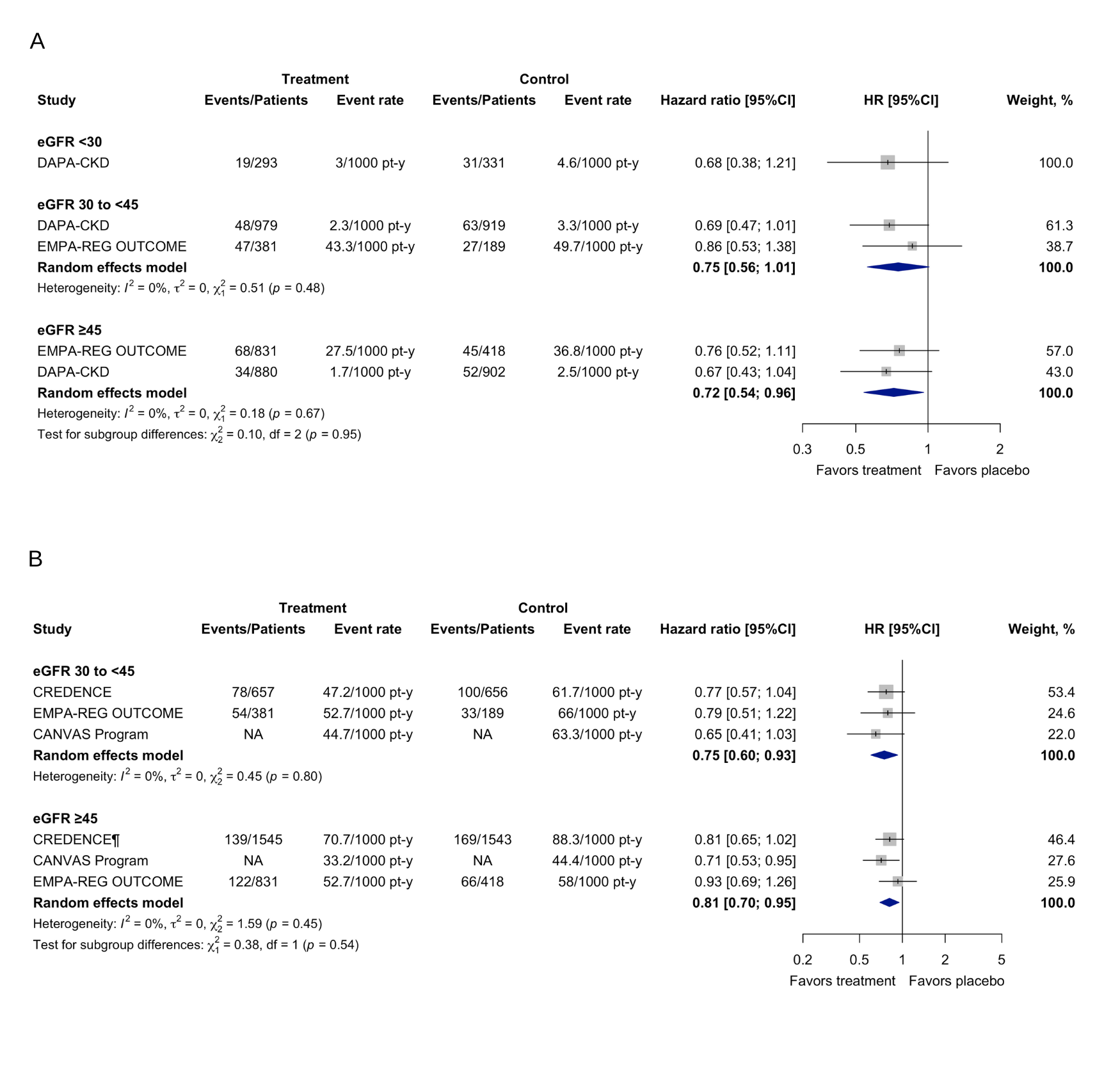

Supplement: S3 Fig — (A) All-cause mortality. (B) MACE outcome. ¶Synthetic estimate created with a fixed effects model meta-analysis. CI, confidence interval; eGFR, estimated glomerular filtration rate; HR, hazard ratio; NA, not available. (TIF) [file pone.0295059.s004.tif]

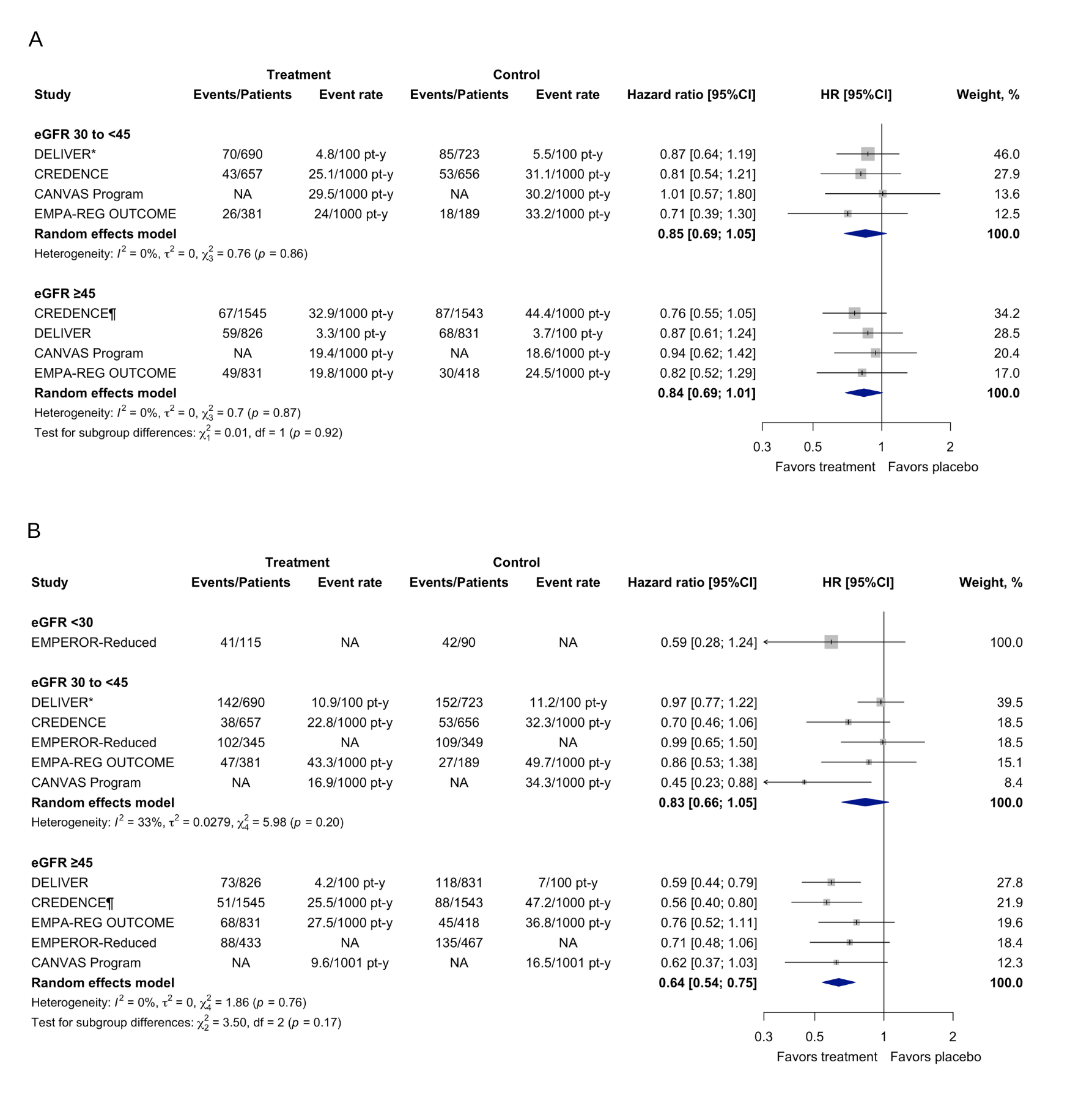

Supplement: S4 Fig — (A) CV death. (B) Hospitalization for HF. *Included patients with an eGFR of 25 to < 45 ml/min/1.73 m2. ¶Synthetic estimate created with a fixed effects model meta-analysis. CI, confidence interval; eGFR, estimated glomerular filtration rate; HR, hazard ratio; NA, not available. (TIF) [file pone.0295059.s005.tif]

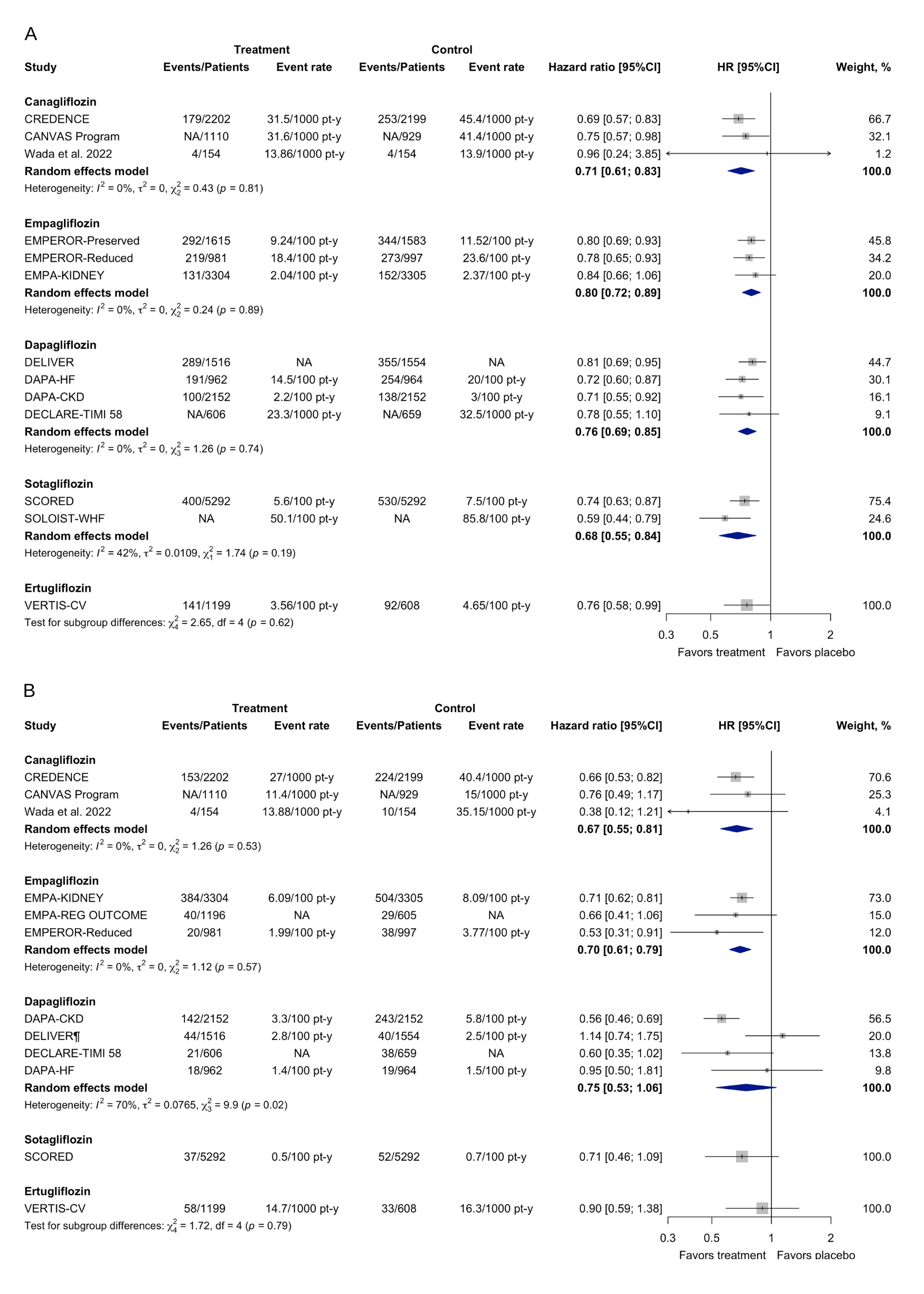

Supplement: S5 Fig — (A) Primary cardiovascular outcome. (B) Primary renal outcome. ¶Synthetic estimate created with a fixed effects model meta-analysis. CI, confidence interval; HR, hazard ratio; NA, not available. (TIF) [file pone.0295059.s006.tif]

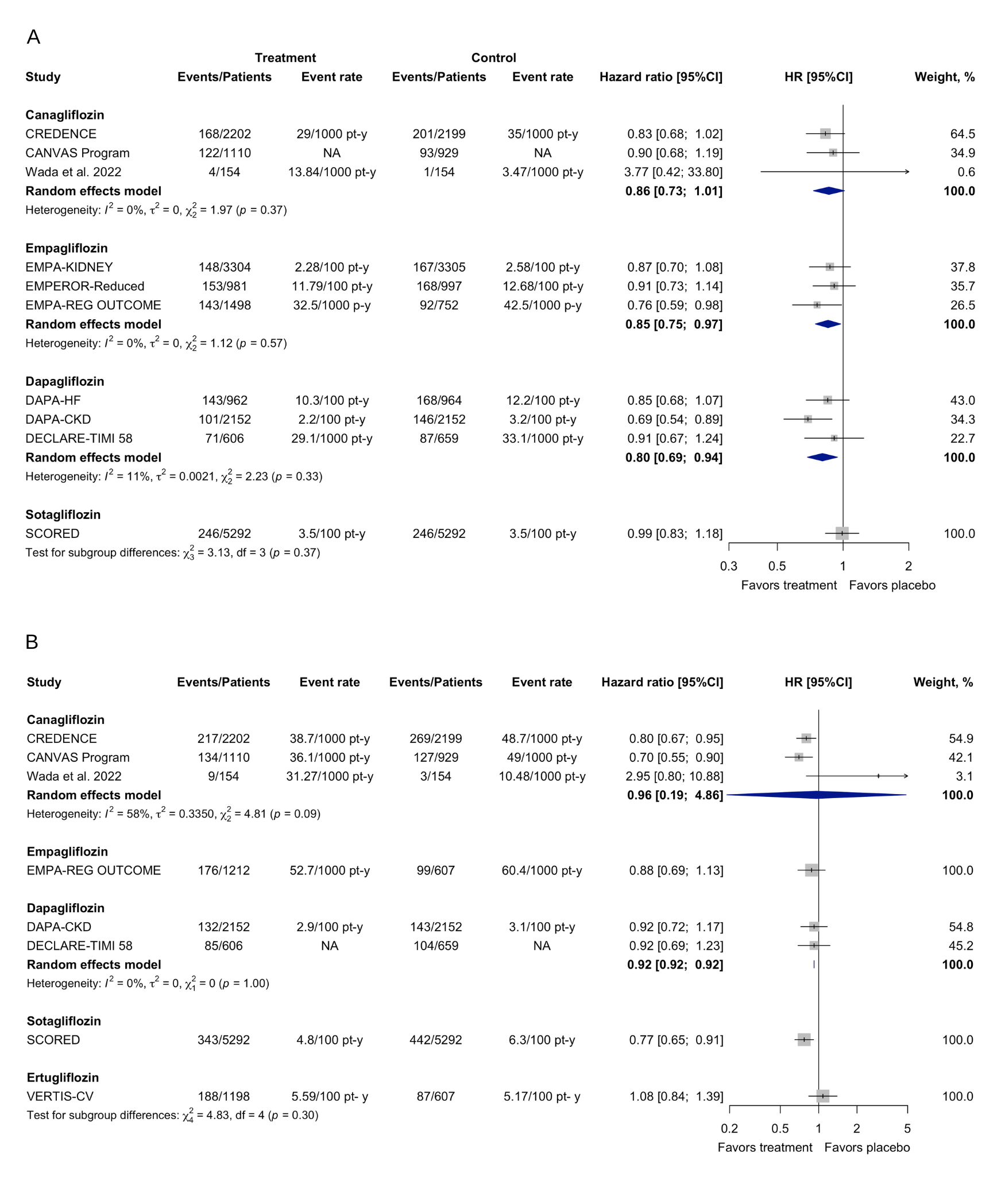

Supplement: S6 Fig — (A) All-cause mortality. (B) MACE outcome. CI, confidence interval; HR, hazard ratio; NA, not available. (TIF) [file pone.0295059.s007.tif]

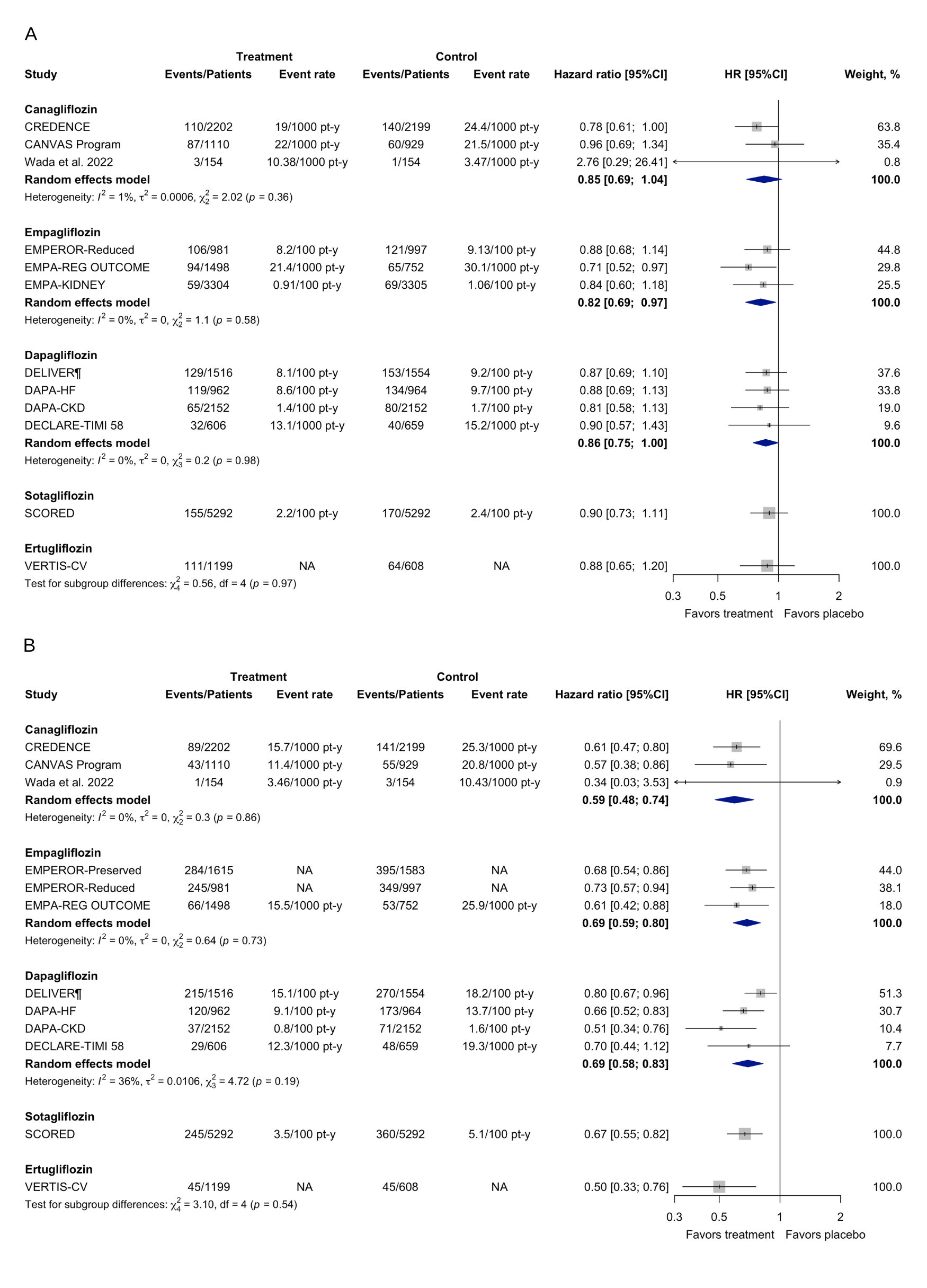

Supplement: S7 Fig — (A) CV death. (B) Hospitalization for HF. ¶Synthetic estimate created with a fixed effects model meta-analysis. CI, confidence interval; HR, hazard ratio; NA, not available. (TIF) [file pone.0295059.s008.tif]

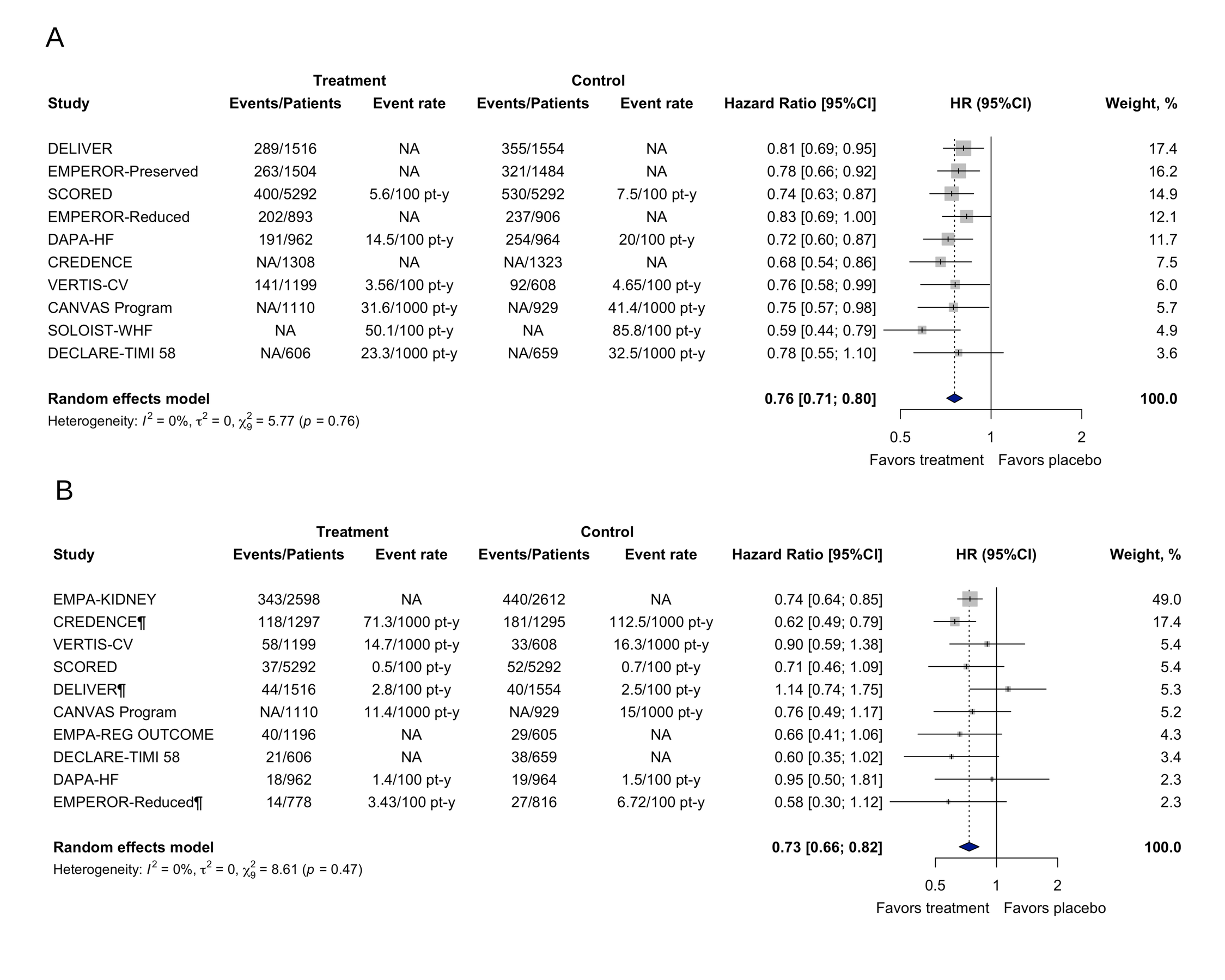

Supplement: S8 Fig — (A) Primary cardiovascular outcome. (B) Primary renal outcome. ¶Synthetic estimate created with a fixed effects model meta-analysis. CI, confidence interval; CKD, chronic kidney disease; HR, hazard ratio; NA, not available. (TIF) [file pone.0295059.s009.tif]

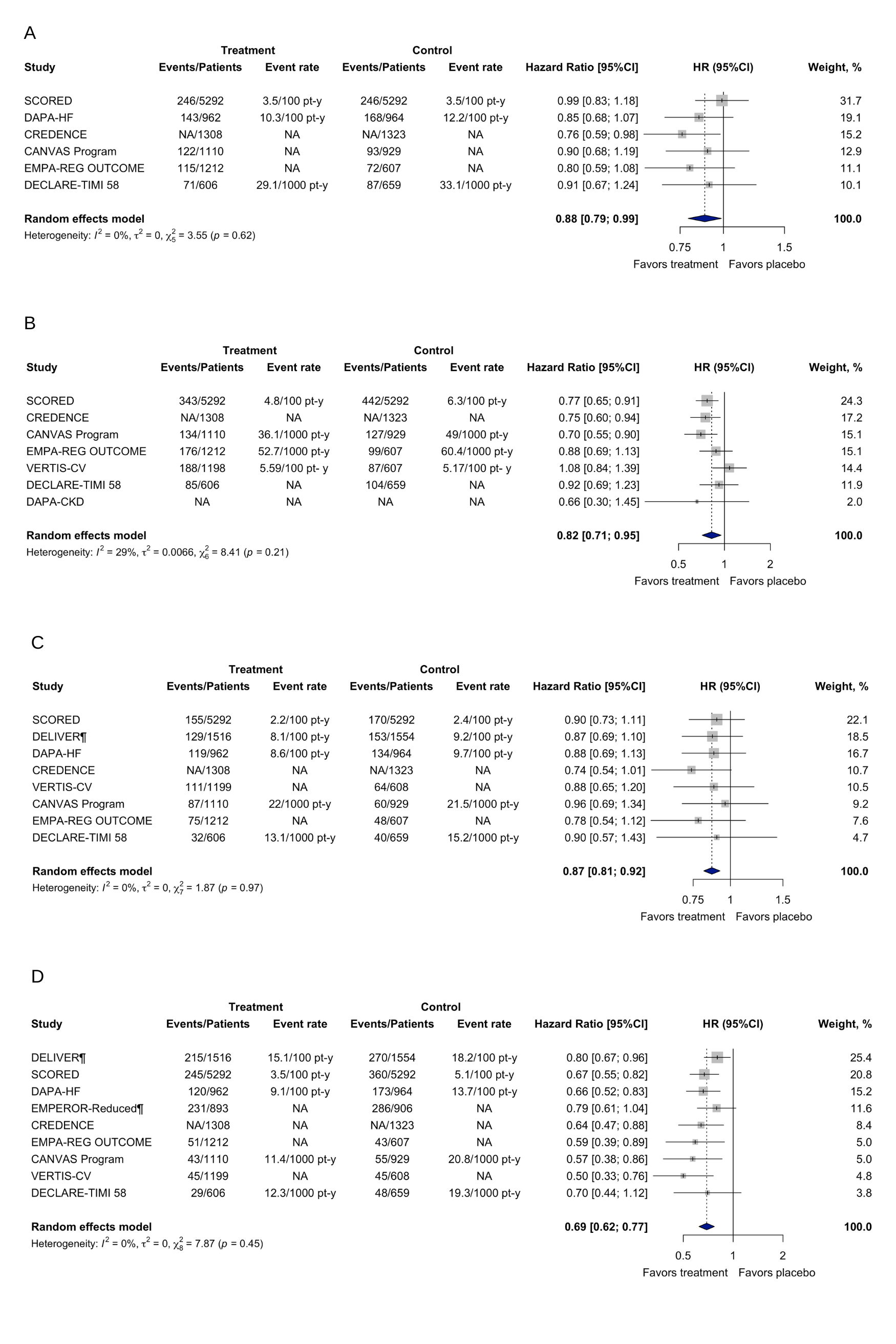

Supplement: S9 Fig — (A) All-cause mortality. (B) MACE outcome. (C) CV death. (D) Hospitalization for HF. ¶Synthetic estimate created with a fixed effects model meta-analysis. CI, confidence interval; CKD, chronic kidney disease; HR, hazard ratio; NA, not available. (TIF) [file pone.0295059.s010.tif]

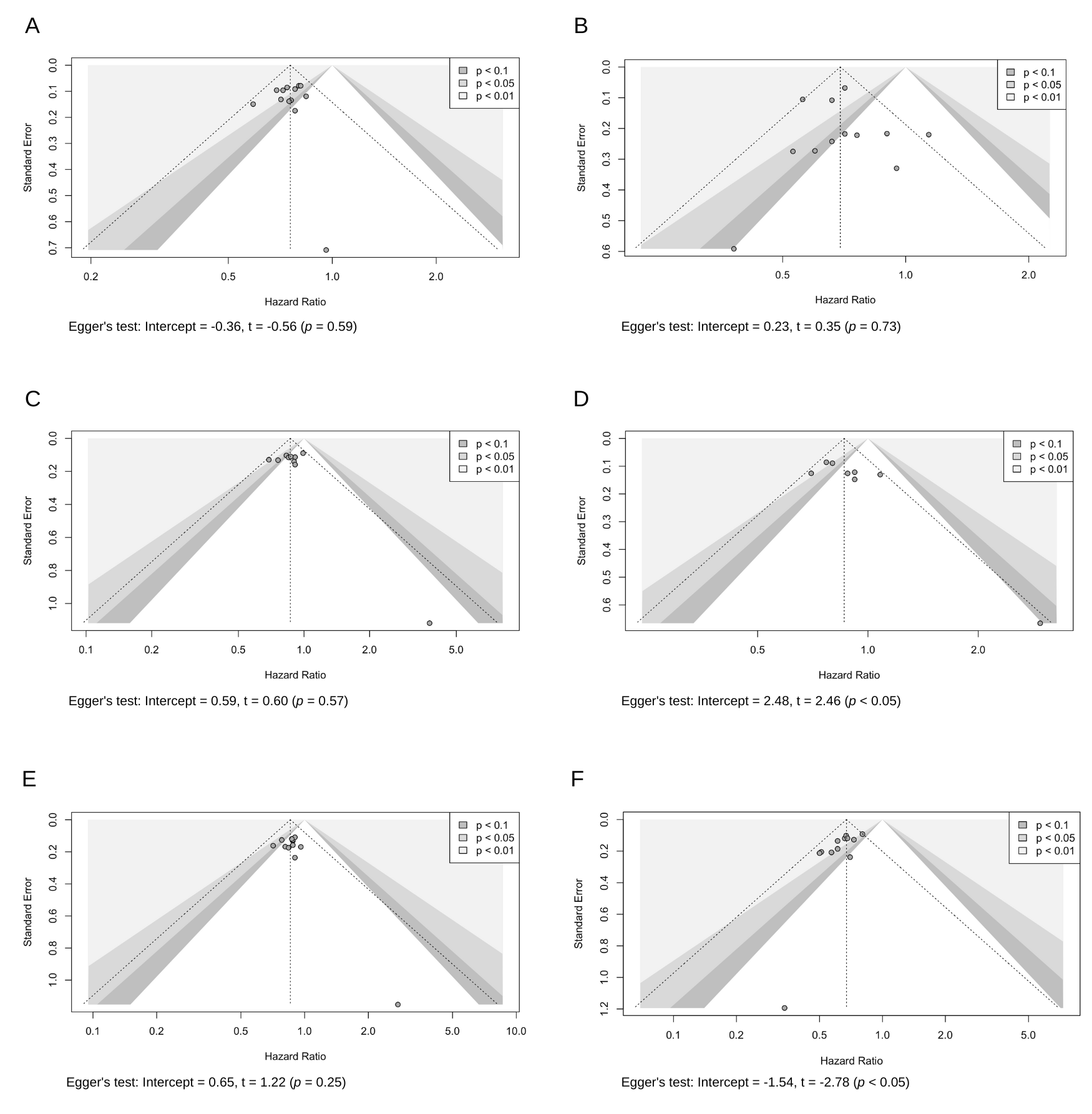

Supplement: S10 Fig — (A) Primary cardiovascular outcome. (B) Primary renal outcome. (C) All-cause mortality. (D) MACE outcome. (E) CV death. (F) Hospitalization for HF. (TIF) [file pone.0295059.s011.tif]
