# Supplementary material for: The effect of sodium-glucose cotransporter 2 inhibitors in patients with chronic kidney disease with or without type 2 diabetes mellitus on cardiovascular and renal outcomes: A systematic review and meta-analysis
Source: PLoS One. 2023 Nov 29;18(11):e0295059. doi: 10.1371/journal.pone.0295059 (PMC10686459; doi:10.1371/journal.pone.0295059)
Supplement: S1 Table — *We used an eGFR < 60 ml/min/1.73 m2 as the definition of chronic kidney disease. CKD, chronic kidney disease; CV, cardiovascular; eGFR, estimated glomerular filtration rate; HF, heart failure; NA, not available; UACR, urine albumin-to-creatinine ratio. (DOCX) [file pone.0295059.s012.docx]

| **Study** | **CKD definition** | **Renal composite outcome** | **Cardiovascular composite outcome** |
| --- | --- | --- | --- |
| EMPEROR-Reduced | eGFR < 60 ml/min/1.73 m^2^ and/or UACR > 300 mg/g | Chronic dialysis or kidney transplant or sustained reduction of ≥40% in eGFR or sustained eGFR (CKD-EPI) <30 ml/min/1.73 m^2^ | Adjudicated hospitalization for HF or CV death |
| CREDENCE | eGRF 30 ml/min/1.73 m2 to < 90 ml/min/1.73 m^2^ AND UACR 300 to 5000 mg/g | End-stage kidney disease, doubling of serum creatinine level, or renal death | CV death or hospitalization for HF |
| DAPA-CKD | eGRF 25 ml/min/1.73 m^2^ to 75 ml/min/1.73 m^2^ AND UACR 200 to 5000 mg/g | Decline in estimated GFR of ≥50%, end-stage kidney disease, or death from renal causes | CV death or hospital admission for HF |
| VERTIS CV | NA* | Doubling of baseline serum creatinine, kidney dialysis/transplant, or renal death | CV death or HF Hospitalization |
| EMPA-REG OUTCOME | NA* | Doubling of serum creatinine level accompanied by eGFR of ≤45 ml/min/1.73 m^2^, initiation of renal-replacement therapy, or death from renal disease | NA |
| DAPA-HF | NA* | ≥50% sustained decline eGFR, End-stage renal disease, Chronic dialysis treatment and renal death | CV death or worsening of HF (HF hospitalization/urgent HF visit) |
| SOLOIST-WHF | NA* | NA | Deaths from CV causes and hospitalizations and urgent visits for HF — total no. of events |
| SCORED | eGRF 25 ml/min/1.73 m^2^ to 60 ml/min/1.73 m^2^ | NA | Total no. or hospitalizations for HF and urgent visits for HF, or death from CV causes |
| CANVAS Program | NA* | 40% reduction in eGFR, kidney failure, or kidney-related death | NA |
| EMPEROR-Preserved | eGFR < 60 ml/min/1.73 m^2^ and/or UACR > 300 mg/g | NA | Adjudicated first hospitalization for HF or CV death |
| DECLARE-TIMI 58 | NA* | Sustained decrease in eGFR by at least 40% to less than 60 mL/min per 1.73 m², end-stage renal disease, or renal death | CV death or hospitalization for HF |
| DELIVER | NA* | ≥50% decline eGFR, End-stage renal disease, or death from kidney causes | CV death or worsening of HF (HF hospitalization/urgent HF visit) |
| EMPA-KIDNEY | eGFR ≥20 to <45 ml/min/1.73 m², regardless of the level of albuminuria OR eGFR of at ≥ 45 to < 90 ml/min/1.73 m² with a UACR ≥ 200 at the screening visit | End-stage kidney disease, Sustained decrease in the eGFR to < 10 ml/min/1.73 m², Sustained decrease in the eGFR ≥ 40%, or death from renal causes | Hospitalization for HF or death from CV causes |
| Wada et al. 2022 | eGRF 30 ml/min/1.73 m2 to < 90 ml/min/1.73 m^2^ AND UACR 300 to 5000 mg/g | End-stage renal disease, doubling of serum creatinine, or renal death | CV death or hospitalization for HF |
