# Supplementary material for: The effect of sodium-glucose cotransporter 2 inhibitors in patients with chronic kidney disease with or without type 2 diabetes mellitus on cardiovascular and renal outcomes: A systematic review and meta-analysis
Source: PLoS One. 2023 Nov 29;18(11):e0295059. doi: 10.1371/journal.pone.0295059 (PMC10686459; doi:10.1371/journal.pone.0295059)
Supplement: S2 Table — (DOCX) [file pone.0295059.s013.docx]

**OVID search strategy**

Database(s): EBM Reviews - Cochrane Central Register of Controlled Trials January 2023, Embase 1974 to 2023 February 23, Ovid MEDLINE(R) and Epub Ahead of Print, In-Process, In-Data-Review & Other Non-Indexed Citations, Daily and Versions 1946 to February 23, 2023

| **#** | **Searches** |
| --- | --- |
| 1 | exp sodium glucose cotransporter 2 inhibitor/ |
| 2 | (Canagliflozin* or Dapagliflozin* or Empagliflozin* or Ertugliflozin* or gliflozin* or Ipragliflozin* or Luseogliflozin* or "SGLT2 inhibitor*" or "SGLT-2 inhibitor*" or "sodium dependent glucose cotransporter 2 inhibitor*" or "sodium glucose cotransporter 2 inhibitor*" or "sodium glucose co-transporter 2 inhibitor*" or Tofogliflozin*).ti,ab,hw,kw. |
| 3 | 1 or 2 |
| 4 | kidney diseases/ or exp "chronic kidney disease-mineral and bone disorder"/ or exp diabetic nephropathies/ or exp renal insufficiency/ |
| 5 | (((kidney* or renal) adj5 (disease* or disorder* or failure* or insufficienc* or nephropath* or outcome* or event* or adverse*)) or "chronic glomerulonephritid*" or CKD or "Diabetic Nephropath*" or esrd).ti,ab,hw,kw. |
| 6 | 4 or 5 |
| 7 | 3 and 6 |
| 8 | exp controlled study/ |
| 9 | exp Randomized Controlled Trial/ |
| 10 | exp triple blind procedure/ |
| 11 | exp Double-Blind Method/ |
| 12 | exp Single-Blind Method/ |
| 13 | exp latin square design/ |
| 14 | exp Placebos/ |
| 15 | exp Placebo Effect/ |
| 16 | randomised controlled trials.sd. |
| 17 | ((control* adj3 study) or (control* adj3 trial) or (randomized adj3 study) or (randomized adj3 trial) or (randomised adj3 study) or (randomised adj3 trial) or "pragmatic clinical trial" or (doubl* adj blind*) or (doubl* adj mask*) or (singl* adj blind*) or (singl* adj mask*) or (tripl* adj blind*) or (tripl* adj mask*) or (trebl* adj blind*) or (trebl* adj mask*) or "latin square" or placebo* or nocebo* or random*).mp,pt. |
| 18 | or/8-17 |
| 19 | 7 and 18 |
| 20 | limit 19 to ("all adult (19 plus years)" or "young adult (19 to 24 years)" or "adult (19 to 44 years)" or "young adult and adult (19-24 and 19-44)" or "middle age (45 to 64 years)" or "middle aged (45 plus years)" or "all aged (65 and over)" or "aged (80 and over)") [Limit not valid in CCTR,Embase; records were retained] |
| 21 | limit 20 to (adult <18 to 64 years> or aged <65+ years>) [Limit not valid in CCTR,Ovid MEDLINE(R),Ovid MEDLINE(R) Daily Update,Ovid MEDLINE(R) PubMed not MEDLINE,Ovid MEDLINE(R) In-Process,Ovid MEDLINE(R) Publisher; records were retained] |
| 22 | limit 19 to ("all infant (birth to 23 months)" or "all child (0 to 18 years)" or "newborn infant (birth to 1 month)" or "infant (1 to 23 months)" or "preschool child (2 to 5 years)" or "child (6 to 12 years)" or "adolescent (13 to 18 years)") [Limit not valid in CCTR,Embase; records were retained] |
| 23 | limit 22 to (embryo or infant or child or preschool child <1 to 6 years> or school child <7 to 12 years> or adolescent <13 to 17 years>) [Limit not valid in CCTR,Ovid MEDLINE(R),Ovid MEDLINE(R) Daily Update,Ovid MEDLINE(R) PubMed not MEDLINE,Ovid MEDLINE(R) In-Process,Ovid MEDLINE(R) Publisher; records were retained] |
| 24 | 23 not 21 |
| 25 | 19 not 24 |
| 26 | (exp animals/ or exp nonhuman/) not exp humans/ |
| 27 | ((alpaca or alpacas or amphibian or amphibians or animal or animals or antelope or armadillo or armadillos or avian or baboon or baboons or beagle or beagles or bee or bees or bird or birds or bison or bovine or buffalo or buffaloes or buffalos or "c elegans" or "Caenorhabditis elegans" or camel or camels or canine or canines or carp or cats or cattle or chick or chicken or chickens or chicks or chimp or chimpanze or chimpanzees or chimps or cow or cows or "D melanogaster" or "dairy calf" or "dairy calves" or deer or dog or dogs or donkey or donkeys or drosophila or "Drosophila melanogaster" or duck or duckling or ducklings or ducks or equid or equids or equine or equines or feline or felines or ferret or ferrets or finch or finches or fish or flatworm or flatworms or fox or foxes or frog or frogs or "fruit flies" or "fruit fly" or "G mellonella" or "Galleria mellonella" or geese or gerbil or gerbils or goat or goats or goose or gorilla or gorillas or hamster or hamsters or hare or hares or heifer or heifers or horse or horses or insect or insects or jellyfish or kangaroo or kangaroos or kitten or kittens or lagomorph or lagomorphs or lamb or lambs or llama or llamas or macaque or macaques or macaw or macaws or marmoset or marmosets or mice or minipig or minipigs or mink or minks or monkey or monkeys or mouse or mule or mules or nematode or nematodes or octopus or octopuses or orangutan or "orang-utan" or orangutans or "orang-utans" or oxen or parrot or parrots or pig or pigeon or pigeons or piglet or piglets or pigs or porcine or primate or primates or quail or rabbit or rabbits or rat or rats or reptile or reptiles or rodent or rodents or ruminant or ruminants or salmon or sheep or shrimp or slug or slugs or swine or tamarin or tamarins or toad or toads or trout or urchin or urchins or vole or voles or waxworm or waxworms or worm or worms or xenopus or "zebra fish" or zebrafish) not (human or humans or patient or patients)).ti,ab,hw,kw. |
| 28 | 25 not (26 or 27) |
| 29 | limit 28 to (editorial or erratum or note or addresses or autobiography or bibliography or biography or blogs or comment or dictionary or directory or interactive tutorial or interview or lectures or legal cases or legislation or news or newspaper article or overall or patient education handout or periodical index or portraits or published erratum or video-audio media or webcasts) [Limit not valid in CCTR,Embase,Ovid MEDLINE(R),Ovid MEDLINE(R) Daily Update,Ovid MEDLINE(R) PubMed not MEDLINE,Ovid MEDLINE(R) In-Process,Ovid MEDLINE(R) Publisher; records were retained] |
| 30 | 28 not 29 |
| 31 | remove duplicates from 30 |

**Scopus search strategy**

1 TITLE-ABS-KEY(Canagliflozin* OR Dapagliflozin* OR Empagliflozin* OR Ertugliflozin* OR gliflozin* OR Ipragliflozin* OR Luseogliflozin* OR "SGLT2 inhibitor*" OR "SGLT-2 inhibitor*" OR "sodium dependent glucose cotransporter 2 inhibitor*" OR "sodium glucose cotransporter 2 inhibitor*" OR "sodium glucose co-transporter 2 inhibitor*" OR Tofogliflozin*)

2 TITLE-ABS-KEY(((kidney* or renal) W/5 (disease* or disorder* or failure* or insufficienc* or nephropath* or outcome* or event* or adverse*)) OR "chronic glomerulonephritid*" OR CKD OR "Diabetic Nephropath*" OR esrd)

3 TITLE-ABS-KEY((control* W/3 study) or (control* W/3 trial) or (randomized W/3 study) or (randomized W/3 trial) or (randomised W/3 study) or (randomised W/3 trial) or "pragmatic clinical trial" or (doubl* W/1 blind*) or (doubl* W/1 mask*) or (singl* W/1 blind*) or (singl* W/1 mask*) or (tripl* W/1 blind*) or (tripl* W/1 mask*) or (trebl* W/1 blind*) or (trebl* W/1 mask*) or "latin square" or placebo* or nocebo* or random*)

4 1 and 2 and 3

5 TITLE-ABS-KEY(newborn* or neonat* or infant* or toddler* or child* or adolescent* or paediatric* or pediatric* or girl or girls or boy or boys or teen or teens or teenager* or preschooler* or "pre-schooler*" or preteen or preteens or "pre-teen" or "pre-teens" or youth or youths) AND NOT TITLE-ABS-KEY(adult or adults or "middle age" or "middle aged" OR elderly OR geriatric* OR "old people" OR "old person*" OR "older people" OR "older person*" OR "very old")

6 4 and not 5

7 TITLE-ABS-KEY((alpaca OR alpacas OR amphibian OR amphibians OR animal OR animals OR antelope OR armadillo OR armadillos OR avian OR baboon OR baboons OR beagle OR beagles OR bee OR bees OR bird OR birds OR bison OR bovine OR buffalo OR buffaloes OR buffalos OR "c elegans" OR "Caenorhabditis elegans" OR camel OR camels OR canine OR canines OR carp OR cats OR cattle OR chick OR chicken OR chickens OR chicks OR chimp OR chimpanze OR chimpanzees OR chimps OR cow OR cows OR "D melanogaster" OR "dairy calf" OR "dairy calves" OR deer OR dog OR dogs OR donkey OR donkeys OR drosophila OR "Drosophila melanogaster" OR duck OR duckling OR ducklings OR ducks OR equid OR equids OR equine OR equines OR feline OR felines OR ferret OR ferrets OR finch OR finches OR fish OR flatworm OR flatworms OR fox OR foxes OR frog OR frogs OR "fruit flies" OR "fruit fly" OR "G mellonella" OR "Galleria mellonella" OR geese OR gerbil OR gerbils OR goat OR goats OR goose OR gorilla OR gorillas OR hamster OR hamsters OR hare OR hares OR heifer OR heifers OR horse OR horses OR insect OR insects OR jellyfish OR kangaroo OR kangaroos OR kitten OR kittens OR lagomorph OR lagomorphs OR lamb OR lambs OR llama OR llamas OR macaque OR macaques OR macaw OR macaws OR marmoset OR marmosets OR mice OR minipig OR minipigs OR mink OR minks OR monkey OR monkeys OR mouse OR mule OR mules OR nematode OR nematodes OR octopus OR octopuses OR orangutan OR "orang-utan" OR orangutans OR "orang-utans" OR oxen OR parrot OR parrots OR pig OR pigeon OR pigeons OR piglet OR piglets OR pigs OR porcine OR primate OR primates OR quail OR rabbit OR rabbits OR rat OR rats OR reptile OR reptiles OR rodent OR rodents OR ruminant OR ruminants OR salmon OR sheep OR shrimp OR slug OR slugs OR swine OR tamarin OR tamarins OR toad OR toads OR trout OR urchin OR urchins OR vole OR voles OR waxworm OR waxworms OR worm OR worms OR xenopus OR "zebra fish" OR zebrafish) AND NOT (human OR humans or patient or patients))

8 6 and not 7

9 DOCTYPE(ed) OR DOCTYPE(bk) OR DOCTYPE(er) OR DOCTYPE(no) OR DOCTYPE(sh)

10 8 and not 9

11 INDEX(embase) OR INDEX(medline) OR PMID(0* OR 1* OR 2* OR 3* OR 4* OR 5* OR 6* OR 7* OR 8* OR 9*)

12 10 and not 11
