# Supplementary material for: The effect of sodium-glucose cotransporter 2 inhibitors in patients with chronic kidney disease with or without type 2 diabetes mellitus on cardiovascular and renal outcomes: A systematic review and meta-analysis
Source: PLoS One. 2023 Nov 29;18(11):e0295059. doi: 10.1371/journal.pone.0295059 (PMC10686459; doi:10.1371/journal.pone.0295059)
Supplement: S3 Table — Data calculated from other publications of the included trial is shaded blue, since it was not directly available in the published articles. ACEI, angiotensin-converting enzyme inhibitors; ARB, angiotensin receptor blockers; CKD, chronic kidney disease; CV, cardiovascular; DPP-4, dipeptidyl peptidase-4; eGFR, estimated glomerular filtration rate; GLP-1, glucose-like peptide-1; HF, Heart failure; IQR, interquartile range; MRA, mineralocorticoid receptor antagonist; n, absolute frequency; NA, not available; SD, standard deviation; SGLT-2, sodium-glucose cotransporter 2; T2DM, type 2 diabetes mellitus; UACR, urine albumin-to-creatinine ratio. (DOCX) [file pone.0295059.s014.docx]

| **Characteristic** | **Study name (n)** | | | | | | | | | | | | | |
| --- | --- | --- | --- | --- | --- | --- | --- | --- | --- | --- | --- | --- | --- | --- |
|  | EMPA-REG OUTCOME (n=2250) | CANVAS Program (n = 2039) | DECLARE-TIMI 58 (n=1265) | CREDENCE (n=4401) | DAPA-HF  (n=1926) | EMPEROR-Reduced (n=1978) | VERTIS CV (n=1807) | DAPA-CKD (n=4304) | SOLOIST-WHF (n= 854) | SCORED (n=10584) | EMPEROR-Preserved (n=3198) | DELIVER (n=3070) | EMPA-KIDNEY (n=6609) | Wada et al. 2022  (n=308) |
| **Year of main publication** | 2015 | 2017 | 2019 | 2019 | 2019 | 2020 | 2020 | 2020 | 2021 | 2021 | 2021 | 2022 | 2022 | 2022 |
| **SGLT-2 inhibitor** | Empagliflozin | Canagliflozin | Dapagliflozin | Canagliflozin | Dapagliflozin | Empagliflozin | Ertugliflozin | Dapagliflozin | Sotagliflozin | Sotagliflozin | Empagliflozin | Dapagliflozin | Empagliflozin | Canagliflozin |
| **Duration of follow-up. median. years** | 3.1 | 2.4 | 4.2 | 2.6 | 1.5 | 1.3 | 3.0 | 2.4 | 0.8 | 1.3 | 2.2 | 2.3 | 2.0 | NA |
| **Included population** |  | | | | | | | | | | | | | |
| Main study population | T2DM and established CV disease | T2DM and atherosclerotic CV disease or CV risk factors | T2DM and established CV disease or CV risk factors | T2DM and CKD | HF with reduced ejection fraction ≤ 40% | HF with reduced ejection fraction ≤ 40% | T2DM and established CV disease | CKD | T2DM and HF hospitalization | T2DM and CKD with CV risk factors | HF with preserved ejection fraction > 40% | HF with preserved ejection fraction > 40% | CKD | T2DM and CKD |
| Inclusion of patients without T2DM | No | No | No | No | Yes | Yes | No | Yes | No | No | Yes | Yes | Yes | No |
| **Patient characteristics** |  | | | | | | | | | | | | | |
| Men | 1562 (69.4) | 1186 (58.2) | 814 (64.3) | 2907 (66.1) | 1392 (72.3) | 1473 (74.5) | 1156 (64) | 2879 (66.9) | NA | 5830 (55.1) | NA | 1564 (50.9) | 4417 (66.8) | 244 (79.2) |
| Women | 688 (30.6) | 853 (41.8) | 451 (35.7) | 1494 (33.9) | 534 (27.7) | 505 (25.5) | 651 (36) | 1425 (33.1) | NA | 4754 (44.9) | NA | 1506 (49.1) | 2192 (33.2) | 64 (20.8) |
| Age. mean (SD) | 66.1 (8.2) | NA | 67.3 (6.6) | 63 (9.2) | NA | 70.2 (9.7) | 68.2 (7.6) | 61.9 (12.1) | NA | NA | NA | 74.5 (9.0) | 63.8 (13.9) | 62.5 (10.7) |
| **Race/ethnicity** |  | | | | | | | | | | | | | |
| White | 1614 (71.7) | 1673 (82.1) | 1088 (86.0) | 2931 (66.6) | NA | 1463 (74.0) | NA | 2290 (53.2) | NA | 8749 (82.7) | NA | 2191 (71.4) | 3859 (58.4) | 0 (0) |
| Asian | 505 (22.4) | 216 (10.6) | NA | 877 (19.9) | NA | 306 (15.5) | NA | 1467 (34.1) | NA | 682 (6.4) | NA | 635 (20.7) | 2393 (36.2) | 308 (100) |
| Black | 108 (4.8) | 46 (2.3) | NA | 224 (5.1) | NA | 115 (5.8) | NA | 191 (4.4) | NA | 364 (3.4) | NA | 77 (2.5) | 262 (4.0) | 0 (0) |
| Other/missing | 23 (1.0) | 103 (5.1) | 177 (14.0) | 369 (8.4) | NA | 94 (4.8) | NA | 356 (8.3) | NA | 789 (7.5) | NA | 167 (5.4) | 95 (1.4) | 0 (0) |
| **Diabetes characteristics** |  | | | | | | | | | | | | | |
| Population with T2DM | 2250 (100) | 2039 (100) | 1265 (100) | 4401 (100) | 982 (51.0) | 1065 (53.8) | 1807 (100) | 2906 (67.5) | 854 (100) | 10584 (100) | NA | 1512 (49.3) | 2936 (44.4) | 308 (100) |
| HbA1C. mean (SD). % | 8.1 (0.9) | NA | 8.2 (1.2) | 8.3 (1.3) | 6.6 (1.4) | NA | 8.2 (0.9) | NA | NA | NA | NA | NA | NA | 7.8 (1.1) |
| **Cardiovascular characteristics** |  | | | | | | | | | | | | | |
| Established cardiovascular disease | 2250 (100) | 1460 (71.6) | NA | 2220 (50.4) | 1926 (100) | 1978 (100) | 1807 (100) | 1610 (37.4) | NA | NA | NA | 3070 (100) | 1765 (26.7) | 308 (100) |
| History of HF | 307 (13.6) | 364 (17.9) | NA | 652 (14.8) | 1926 (100) | 1978 (100) | NA | 468 (10.9) | NA | 3283 (31.0) | NA | 3070 (100) | NA | NA |
| **Renal characteristics** |  | | | | | | | | | | | | | |
| eGFR < 60 ml/min/1.73m2 | 1819 (80.8) | 2039 (100) | 1265 (100) | 2592 (58.9) | 1926 (100) | 1799 (91.0) | 1807 (100) | 3850 (89.5) | NA | 10584 (100) | NA | 3070 (100) | 5210 (78.8) | 180 (58.4) |
| Urine ACR ≥ 300 mg/g | 769 (34.2) | NA | 167 (13.5) | 3371 (76.6) | NA | NA | NA | NA | NA | 3286 (31.0) | NA | NA | 3417 (51.7) | NA |
| **Cardiovascular medications** |  | | | | | | | | | | | | | |
| ACEI or ARB blockade | 1892 (84.1) | 1655 (81.2) | 1097 (86.7) | 4395 (99.9) | 1542 (80.1) | 1320 (66.7) | 1503 (83.2) | 4224 (98.1) | NA | 9229 (87.2) | NA | 2178 (70.9) | 5628 (85.2) | NA |
| β-Blocker | 1495 (66.4) | 1275 (62.5) | 816 (64.5) | 1770 (40.2) | 1838 (95.4) | 1875 (94.8) | NA | NA | NA | 6616 (62.5) | NA | 2496 (81.3) | NA | NA |
| Statin/other lipid lowering drugs | 1755 (78.0) | 1590 (78.0) | 1031 (81.5) | 3036 (69.0) | NA | 1455 (73.6) | 1575 (87.2) | 2794 (64.9) | NA | NA | NA | NA | 4378 (66.2) | NA |
| Diuretics (other than MRA) | 1240 (55.1) | 1222 (59.9) | 773 (61.1) | 2057 (46.7) | 1835 (95.3 | 1790 (90.5) | 1020 (56.4) | 1882 (43.7) | NA | NA | NA | 2492 (81.2) | 2815 (42.6) | NA |
| **Antihyperglycemic medication** |  | | | | | | | | | | | | | |
| Insulin | 1306 (58.0) | 1244 (61.0) | 711 (56.2) | 2884 (65.5) | 304 (15.8 | NA | 1059 (58.6) | 1598 (37.1) | NA | 6722 (63.5) | NA | NA | NA | NA |
| Metformin | 1412 (62.8) | 1162 (57.0) | 843 (66.6) | 2545 (57.8) | 406 (21.1) | NA | 1127 (62.4) | 1242 (28.9) | NA | 5862 (55.4) | NA | NA | NA | NA |
| Sulfonylurea | 884 (39.3) | 758 (37.2) | 445 (35.2) | 1268 (28.8) | 198 (10.3) | NA | NA | 774 (18.0) | NA | 2886 (27.3) | NA | NA | NA | NA |
| DPP-4 inhibitor | NA | 283 (13.9) | 191 (15.1) | 751 (17.1) | 164 (8.5) | NA | NA | 742 (17.2) | NA | 2085 (19.7) | NA | NA | NA | NA |
| GLP-1 receptor agonist | NA | 83 (4.1) | 72 (5.7) | 183 (4.2) | 15 (0.8) | NA | NA | 122 (2.8) | NA | 663 (6.0) | NA | NA | NA | NA |
