# Supplementary material for: The effect of sodium-glucose cotransporter 2 inhibitors in patients with chronic kidney disease with or without type 2 diabetes mellitus on cardiovascular and renal outcomes: A systematic review and meta-analysis
Source: PLoS One. 2023 Nov 29;18(11):e0295059. doi: 10.1371/journal.pone.0295059 (PMC10686459; doi:10.1371/journal.pone.0295059)
Supplement: S4 Table — (DOCX) [file pone.0295059.s015.docx]

| **SGLT-2 inhibitors and standard therapy compared to standard therapy alone for chronic kidney disease** | | | | | | | | | | | |
| --- | --- | --- | --- | --- | --- | --- | --- | --- | --- | --- | --- |
| **Certainty assessment** | | | | | | | **Summary of findings** | | | | |
| **Participants** **(studies)** | **Risk of bias** | **Inconsistency** | **Indirectness** | **Imprecision** | **Publication bias** | **Overall certainty of evidence** | **Study event rates (%)** | | **Relative effect (95% CI)** | **Anticipated absolute effects** | |
|  |  |  |  |  |  |  | **With placebo and standard therapy alone** | **With SGLT-2 inhibitors and standard therapy** |  | **Risk with placebo and standard therapy alone** | **Risk difference with SGLT-2 inhibitors and standard therapy** |
| **Primary cardiovascular outcome** | | | | | | | | | | | |
| 41489 (13 RCTs)^a,b^ | Not serious | Not serious | Serious^c^ | Not serious | None | ⨁⨁⨁◯ Moderate | 2395/20396 (11.7%) ^a,b^ | 1946/21093 (9.2%) ^a,b^ | **HR 0.76** (0.72 to 0.79) | 117 per 1,000^a,b^ | **27 fewer per 1,000** (from 31 fewer to 23 fewer) |
| **Primary renal outcome** | | | | | | | | | | | |
| 40092 (12 RCTs)^d^ | Not serious | Not serious | Serious^c^ | Not serious | None | ⨁⨁⨁◯ Moderate | 1230/19418 (6.3%) ^d^ | 921/20674 (4.5%) ^d^ | **HR 0.69** (0.61 to 0.79) | 63 per 1,000^d^ | **19 fewer per 1,000** (from 24 fewer to 13 fewer) |
| **All-cause mortality** | | | | | | | | | | | |
| 35664 (10 RCTs) | Not serious | Not serious | Serious^c^ | Not serious | None | ⨁⨁⨁◯ Moderate | 1369/17403 (7.9%) | 1299/18261 (7.1%) | **HR 0.87** (0.79 to 0.94) | 79 per 1,000 | **10 fewer per 1,000** (from 16 fewer to 5 fewer) |
| **Major adverse cardiovascular events** | | | | | | | | | | | |
| 26525 (8 RCTs) | Not serious | Not serious | Serious^c^ | Not serious | None^e^ | ⨁⨁⨁◯ Moderate | 1274/12599 (10.1%) | 1284/13926 (9.2%) | **HR 0.86** (0.74 to 1.01) | 101 per 1,000 | **14 fewer per 1,000** (from 25 fewer to 1 more) |
| **Cardiovascular death** | | | | | | | | | | | |
| 40541 (12 RCTs) | Not serious | Not serious | Serious^c^ | Not serious | None | ⨁⨁⨁◯ Moderate | 1097/19565 (5.6%) | 1070/20976 (5.1%) | **HR 0.86** (0.81 to 0.91) | 56 per 1,000 | **8 fewer per 1,000** (from 10 fewer to 5 fewer) |
| **Hospitalization for heart failure** | | | | | | | | | | | |
| 37130 (12 RCTs) | Not serious | Not serious | Serious^c^ | Not serious | None^e^ | ⨁⨁⨁◯ Moderate | 1963/17843 (11.0%) | 1419/19287 (7.4%) | **HR 0.67** (0.62 to 0.73) | 110 per 1,000 | **35 fewer per 1,000** (from 40 fewer to 28 fewer) |

**CI:** confidence interval; **HR:** hazard Ratio

#### Explanations

a. The number of participants was unavailable for the SOLOIST-WHF trial. Those patients were not included in the total number.

b. The number of events was unavailable for the SOLOIST-WHF, DECLARE-TIMI 58 and CANVAS Program trials. Those events were not included in the total number.

c. Most of the studies were designed to evaluate other diseases such as T2DM and HF; and CKD was a subgroup of the sample.

d. The number of events was unavailable for the CANVAS Program trial. Those events were not included in the total number.

e. The Egger´s test showed funnel plot asymmetry for this outcome. However, the overall suspicion of publication bias was not strong.
