# Supplementary material for: The effect of sodium-glucose cotransporter 2 inhibitors in patients with chronic kidney disease with or without type 2 diabetes mellitus on cardiovascular and renal outcomes: A systematic review and meta-analysis
Source: PLoS One. 2023 Nov 29;18(11):e0295059. doi: 10.1371/journal.pone.0295059 (PMC10686459; doi:10.1371/journal.pone.0295059)
Supplement: Attachment — Submitted filename: Manuscript_changescontrol.docx [file pone.0295059.s017.docx]

**The effect of sodium-glucose cotransporter 2 inhibitors in patients with chronic kidney disease with or without type 2 diabetes mellitus on cardiovascular and renal outcomes: a systematic review and meta-analysis**

**Short title: Sodium-glucose cotransporter 2 inhibitors in patients with chronic kidney disease**

Carlos Ignacio Reyes-Farias^1¶^, Marcelo Reategui-Diaz^1¶*^, Franco Romani-Romani^1^, Larry Prokop^2^

^1^ Faculty of Medicine, Universidad de Piura, Lima, Lima, Peru

^2^ Knowledge and Evaluation Research Unit, Mayo Clinic, Rochester, USA

* Corresponding author

E-mail: [fabio.reategui@alum.udep.edu.pe](mailto:fabio.reategui@alum.udep.edu.pe) (MR)

^¶^These authors contributed equally to this work.

# Abstract

**Background:** Sodium-glucose cotransporter 2 (SGLT-2) inhibitors have shown a favorable effect on cardiovascular and renal outcomes in patients with type 2 diabetes mellitus (T2DM). However, their efficacy in patients with chronic kidney disease (CKD) with or without T2DM has not yet been analyzed.

**Objective:** To assess the cardiovascular and renal effects of SGLT-2 inhibitors in patients with CKD with and without T2DM.

**Methods:** We searched MEDLINE, EMBASE, CENTRAL and Scopus for randomized controlled trials of SGLT-2 inhibitors that evaluated cardiovascular and kidney outcomes in patients with CKD. We defined 2 primary outcomes: a composite of cardiovascular death or hospitalization for heart failure, and a composite renal outcome. For each outcome, we obtained overall hazard ratios with 95% confidence intervals by using a random effects model.

**Results:** We included 13 randomized controlled trials. SGLT-2 inhibitors decreased the hazard for the primary cardiovascular outcome (HR 0.76; [95% CI 0.72-0.79]) and the primary renal outcome (HR 0.69; [95% CI 0.61-0.79]) in patients with CKD with or without T2DM. On the subgroup analyses, we did not find significant differences between diabetic and nondiabetic patients.

**Conclusion:** In patients with CKD, treatment with SGLT-2 inhibitors in addition to standard therapy conferred protection against cardiovascular and renal outcomes. Further research on patients with non-diabetic CKD should be done to confirm the utility of these medications in this population. (PROSPERO ID: CRD42021275012)

**Key words:** Renal Insufficiency, Chronic; Sodium-Glucose Transporter 2 Inhibitors; Meta-Analysis

# Introduction

Chronic kidney disease (CKD) is an important cause of mortality worldwide [1]. Until recently, only angiotensin-converting enzyme (ACE) inhibitors and angiotensin-receptor blockers (ARBs) had been shown to decrease proteinuria and prevent progression to end-stage kidney disease [2,3].

Sodium-glucose cotransporter 2 (SGLT-2) inhibitors are used to treat people with type 2 diabetes mellitus (T2DM) [4,5]. They control glycemia and HbA1c by inhibiting the SGLT-2 cotransporter present in proximal tubules, responsible for the reabsorption of the majority of the filtered glucose in the glomeruli. However, SGLT-2 inhibitors have other non-glycemic effects, such as lowering glomerular hyperfiltration and proteinuria, and anti-inflammatory properties in the glomeruli [6–9]. The relevance of these mechanisms has been reflected in the DAPA-CKD [10] and EMPA-KIDNEY [11] trials, which included both diabetic and non-diabetic patients. These trials have shown a favorable effect of the use of SGLT-2 inhibitors on cardiovascular and renal outcomes. This may suggest that these drugs could benefit patients with chronic kidney disease (CKD) in these outcomes, whether the kidney injury is associated with T2DM or not.

Previous systematic reviews have demonstrated the efficacy of SGLT-2 inhibitors in reducing the risk of cardiovascular and renal events in patients with CKD and T2DM [12–15]. One systematic review included studies in both diabetic and non-diabetic CKD patients [16]. However, they analyzed the data from trials that included only CKD patients and did not consider the data from trials in which CKD patients were a subgroup. Thus, whether SGLT-2 inhibitors are efficacious in the subgroups of CKD patients with or without T2DM was not evaluated across all trials.

The effect of SGLT-2 inhibitors on cardiovascular and renal outcomes in patients with CKD with or without T2DM has not been yet established. Assessing the benefits of SGLT-2 inhibitors in people with CKD regardless of the presence of T2DM may help clarify a new therapeutic role for this drug. The aim of this systematic review was to assess the cardiovascular and renal effects of SGLT-2 inhibitors in patients with CKD and to evaluate whether the presence of T2DM modifies their overall effect.

# Methods

To conduct this study, we followed the Preferred Reporting Items for Systematic Reviews and Meta-analyses (PRISMA) 2020 guideline [17]. The protocol of this systematic review and meta-analysis has been registered in PROSPERO (CRD42021275012).

## Eligibility criteria

We included randomized controlled trials of SGLT-2 inhibitors that evaluated cardiovascular and kidney outcomes in patients with CKD. There were no restrictions regarding the type of SGLT-2 inhibitor,and the comparator group could receive either placebo and standard therapy or standard therapy alone.Patients needed to be adults with CKD, regardless of the definition of CKD, and with or without T2DM. Studies were included regardless of differences in time to follow-up.

We defined two primary outcomes. First, the cardiovascular composite outcome included hospitalization for heart failure (HF) or cardiovascular (CV) death. Second, a renal composite outcome was defined as doubling of serum creatinine, decline of eGFR > 50%, end-stage kidney disease, renal replacement therapy, transplantation, or renal death. The different definitions of primary outcomes are presented in the Supplement (S1 Table). The secondary outcomes were all-cause mortality; major adverse cardiovascular events (MACE, which consists of non-fatal stroke, non-fatal myocardial infarction and CV death); hospitalization for HF; and CV death. We included the information of interest from studies reporting subgroups that met our inclusion criteria.

There were studies reporting subgroups that met our inclusion criteria. These were included but only the information of interest was extracted and used in this study.

## Information sources and search strategy

A comprehensive search from each database's inception to February 24th, 2023 was conducted with no language restriction. The search was later updated to February 24th, 2023, and the retrieved records underwent the same selection process as the original search results. The databases included Ovid MEDLINE(R) and Epub Ahead of Print, In-Process & Other Non-Indexed Citations, and Daily, Ovid EMBASE, Ovid Cochrane Central Register of Controlled Trials (CENTRAL), and Scopus. The search strategy is detailed in the Supplement (S2 Table). We searched for additional records in the references section of the selected studies.

## Screening of studies

Before the selection process, we performed a pilot test of the eligibility criteria to reach consistency between reviewers. The abstract screening and the full-text assessment for eligibility was done independently by two authors (C.R., M.R.) with DistillerSR Version 2.40.0 (Evidence Partners; 2022). The final decision was confirmed by a third person (O.J.P.) in case of disagreement.

## Data extraction

### Data collection and author contact

Data extraction was performed independently and individually by C.R. and M.R. using Microsoft® Excel® Version 2208. Then, both authors crosschecked each other’s extracted information to ensure consistency; if a disagreement was found, both reviewed the full-text article again. We requested unreported outcome data from the corresponding authors of each study and included the information they provided us in the analyses. We extracted the corresponding authors’ information and contacted them via e-mail once. Out of 15 emails sent, seven authors responded. Three authors shared published research papers which we had not retrieved with our search strategy.

### Data items

Two authors independently extracted the following data of each study: publication year, trial registration number, inclusion and exclusion criteria, SGLT-2 inhibitor type, comparator type (placebo or standard care) and follow-up time (years), patient baseline characteristics and clinical outcomes (primary and secondary outcomes as described above), and risk of bias indicators (shown in the section below). The baseline characteristics were sex, age (years), race, diabetes status (T2DM, no T2DM) , diabetes medications (insulin, metformin, sulfonylurea, DPP-4 inhibitor, GLP-1 receptor agonist), HbA1C (percentage), CV history (any CV disease and HF) and CV medications (ACE inhibitors or ARB, β-Blocker, statin and diuretic), and renal baseline characteristics (estimated glomerular filtration rate [eGFR] < 60 ml/min/1.73m2 and urine albumin-to-creatinine ratio [UACR] ≥ 300 mg/g). We only considered the data from the patients with CKD according to the definition of each study and/or patients with an eGFR < 60 ml/min/1.73 m^2^ (S1 Table).

## Risk of bias assessment

Study quality was assessed independently and individually by C.R. and M.R. using the Cochrane Risk of Bias Tool 2. This tool consists of five domains (randomization process, deviations from the intended interventions, missing outcome data, measurement of the outcome, and selection of the reported result) [18].

We used the two primary outcomes for our evaluation and rated the overall risk of bias of each study as ‘low’, ‘some concerns’, or ‘high’. Any disagreement was discussed between the authors to reach a consensus. We generated summary charts with the web-based version of the *robvis* package [19].

## Quality of the evidence

Two authors (C.R., M.R.) independently assessed the quality of the evidence using the Grading of Recommendations Assessment, Development, and Evaluation (GRADE) system [20]. Disagreements were resolved by discussion.

The GRADE system consists of five domains (risk of bias, imprecision, inconsistency, indirectness, and publication bias) to assess the level of certainty of the evidence for each outcome as high, moderate, low, or very low. We considered a large magnitude of effect (Hazard ratio [HR] < 0.5), a dose-response gradient and a plausible confounding as criteria to increase the level of certainty [21].

We used the GRADEpro GDT software to elaborate ‘Summary of the evidence’ table [22]. All the reasons to rate up or down the quality of the evidence were documented as footnotes.

## Statistical analysis

Patient baseline characteristics were summarized according to the type of variable. For qualitative variables, the frequency and percentage were reported; for quantitative variables, the mean and the standard deviation were used instead. Ifdata was not directly available for the subgroup of all CKD patients in a study, we calculated it from the separate intervention and control groups. For the qualitative variables, we added up the frequencies and calculated the percentages based on the total number of participants. For the quantitative variables, we calculated the weighted means and weighted standard deviations, considering the intervention and control groups sizes.

Time-to-event CV and renal outcomes are presented as incidence rate (events per 100 person-years or events per 1000 person-years), and when appropriate, as cumulative incidence (events/total participants). All outcomes were summarized by using the reported HR and their corresponding 95% confidence intervals (CI) in each study.

We performed a meta-analysis including all studies with available data. We fit the random effects model with the Paule-Mandel estimation of the tau-squared [23], since it was more appropriate for binary outcomes than the restricted maximum likelihood method we originally considered. The heterogeneity was adjusted with the Hartung-Knapp method which accounts for the uncertainty in the estimate of tau-squared [24]. The I-squared statistic was calculated to assess the percentage of total variability attributed to heterogeneity among the true effects in our meta-analysis [25].

When a study reported effect estimates (HR) for complementary subgroups from within an outcome such as varying levels of eGFR (45 to < 60 ml/min/1.73 m^2^ and eGFR < 45 ml/min/1.73 m^2^), we aggregated these subgroups with a fixed effects model as described by Borenstein *et al.* [26]. For instance, based on our example a new HR was generated for patients with eGFR < 60ml/min/1.73 m^2^.

We performed subgroup analyses to detect differences in the effect of SGLT-2 inhibitors in patients according to the presence of T2DM. We assessed the interaction between the two subgroups with a Wald-type test. We also calculated the difference between effect sizes and their corresponding 95% confidence intervals [27].

For each analysis we used the most comprehensive definition of CKD available in each study, which could include patients with either a reduced eGFR or significant albuminuria (S2 Table). If this definition was not provided, we established an eGFR < 60 ml/min/1.73 m^2^ as diagnostic of CKD and included these patients in the meta-analysis.

Although this analysis was not described in our protocol, if the Hartung-Knapp adjustment was suspected to be interfering with the precision of a confidence interval, we performed a sensitivity analysis without the adjustment to confirm the result. Additionally, we performed a sensitivity analysis to re-assess our main meta-analysis only in patients with CKD defined as eGFR < 60 ml/min/1.73 m^2^.

Publication bias was explored using contour enhanced funnel plots for the primary and secondary outcomes. Additionally, we performed the Egger’s regression test to statistically assess the risk of publication bias [28]. We originally intended to explore publication bias in outcomes with 10 or more studies; however, the large sample size of the included trials allowed us to evaluate all outcomes.

All the analyses were done with R version 4.2.1 [29] and RStudio version 2022.07.1+554 [30] using the *meta* package [31].

# Results

## Study selection

We retrieved 5009 records from databases. After duplicate removal (n= 1534) and abstract screening (n= 3475) we assessed 250 full-text articles for eligibility. Of these, 24 articles that corresponded to 13 clinical trials were included [10,11,32–53].

We searched for other documents by reviewing citations from included articles. With this strategy we identified three articles with additional outcome data from included trials [54–56], as well as two articles from a trial not previously found [57,58]. Furthermore, we obtained five articles from included trials through author contact [13,15,59–61].In total, we included 34 articles from 14 trials (Fig 1).

**Fig 1. PRISMA Flow chart.** *Wrong study design includes articles of rationale and protocols; **^¶^**Wrong population includes articles of studies that analyzed subgroups of the trial sample that were not of interest for our review.

## Baseline characteristics

We included 44 593 patients from 14 randomized placebo-controlled clinical trials [10,11,32–40,52,56,57] of five SGLT-2 inhibitors (empagliflozin [n=4], canagliflozin [n=3], dapagliflozin [n=4], ertugliflozin [n=1], and sotagliflozin [n=2]) in patients with CKD. S3 Table shows the characteristics of the patients.

Out of the 14 studies included in the quantitative synthesis, five included only patients with CKD, and nine also included non-CKD patients as well. For the latter we only extracted the data from the CKD subgroups. Furthermore, six studies included patients with and without T2DM, while eight only considered patients with T2DM. Finally, only two studies compared patients with CKD and T2DM with patients with CKD without T2DM. For the subgroup analysis, except for the primary renal outcome, we only used data from the DAPA-CKD trial for the non-diabetic subgroups, since there was no other study that reported this data. The median follow-up ranged from 0.8 to 4.2 years across all the trials.

## Risk of bias

The overall risk of bias was judged as ‘low’ in eight trials, ‘some concerns’ in six trials, and no trials were judged as ‘high’ risk of bias. Of the trials with ‘some concerns’, five of them had unclear information in the selection of the reported result and one had deviations from intended intervention. Additionally, the CANVAS Program did not make an adequate statement about missing outcome data neither in any of publications nor the protocol (S1 Fig).

## Quality of the evidence

The ‘Summary of the evidence’ table showed moderate certainty of evidence for most of the outcomes, except for the MACE and hospitalization for HF outcomes, which were rated as low quality of evidence due to concerns of indirectness and publication bias. Indirectness was deemed as ‘serious’ for every outcome, as most of the studies were designed to evaluate diseases other than CKD such as T2DM and HF (S4 Table).

## Main analysis

### Primary outcomes

SGLT-2 inhibitors decreased the hazard for the primary CV outcome in patients with CKD regardless of the presence of T2DM (HR 0.76; [95% CI 0.72-0.79]; τ^2^ = 0; I^2^ = 0%). Likewise, SGLT-2 inhibitors significantly reduced the occurrence of the primary renal outcome (HR 0.69; [95% CI 0.61-0.79]; τ^2^ = 0.0093; I^2^ = 23%) (Fig 2).

**Fig 2. Effects of SGLT-2 inhibitors on the primary outcomes in patients with CKD with or without T2DM.** (A) Primary cardiovascular outcome. (B) Primary renal outcome. ^¶^Effect estimate calculated with a fixed effects model meta-analysis. CI, confidence interval; HR, hazard ratio; NA, not available.

### Secondary outcomes

In patients with CKD with or without T2DM, SGLT-2 inhibitors decreased the risk for all-cause mortality (HR 0.87; [95% CI 0.79-0.94]; τ^2^ = 0; I^2^ = 0%), CV death (HR 0.86; [95% CI 0.81-0.91]; τ^2^ = 0; I^2^ = 0%), and hospitalization for HF (HR 0.67; [95% CI 0.62-0.73]; τ^2^ = 0 ; I^2^ = 0%) (Fig 3). However, SGLT-2 inhibitors did not reduce the risk for the MACE outcome (HR 0.86; [95% CI 0.74-1.01]; τ^2^ = 0.0178; I^2^ = 41%).

**Fig 3. Effects of SGLT-2 inhibitors on the secondary outcomes in patients with CKD with or without T2DM.** (A) All-cause mortality. (B) MACE. (C) CV death. (D) Hospitalization for HF. ^¶^Synthetic estimate created with a fixed effects model meta-analysis. CI, confidence interval; HR, hazard ratio; NA, not available.

## Subgroup analysis

The subgroup analyses according to the presence of T2DM are shown in Figs 4 and 5. We did not find significant differences between diabetic and nondiabetic patients in any outcome. We found a significant reduction of the hazard for the primary renal outcome in the subgroup of patients with T2DM (HR 0.65; [95% CI 0.59-0.72]; τ^2^ = 0; I^2^ = 0%). Although this protective effect was also found in the subgroup of patients without T2DM, this association was not significant, and pooled studies were heterogenous (HR 0.66; [95% CI 0.04-11.16]; τ^2^ = 0.0764; I^2^ = 75%).

**Fig 4. Effects of SGLT-2 inhibitors on the primary outcomes in patients with CKD according to T2DM status.** (A) Primary cardiovascular outcome. (B) Primary renal outcome. CI, confidence interval; HR, hazard ratio; NA, not available; T2DM, type 2 diabetes mellitus.

**Fig 5. Effects of SGLT-2 inhibitors on the secondary outcomes in patients with CKD according to T2DM status.** (A) All-cause mortality. (B) MACE outcome. (C) CV death. (D) Hospitalization for HF. ^¶^Synthetic estimate created with a fixed effects model meta-analysis. CI, confidence interval; HR, hazard ratio; NA, not available; T2DM, type 2 diabetes mellitus.

## Sensitivity analysis

We decided to perform the subgroup analysis for the primary renal outcome without the Hartung-Knapp adjustment, as it seemed to artificially increase the confidence interval.We found a narrower but still not statistically significant confidence interval for this subgroup (HR 0.66; [95% CI 0.43-1.02]; τ^2^ = 0.0764; I^2^ = 75%), and the test for subgroup differences remained not significant (*p* = 0.93).

The effect of SGLT-2 inhibitors when only considering patients with an eGFR < 60 ml/min/1.73 m^2^ were consistent with the main analyses for the primary and secondary outcomes, except for the MACE outcome. The results of this sensitivity analysis are presented in the Supplement (S2 and S3 Figs).

## Publication bias assessment

We only found evidence of publication bias in the MACE and hospitalization for HF outcomes (*p* < 0.05) (S4 Fig). With regards to the availability of data, we were only able to extract data of patients with CKD without T2DM from 2 studies [10,11]. The other studies that included patients with CKD without T2DM did not report outcome data for this specific subgroup.

# Discussion

In this systematic review and meta-analysis, moderate quality of evidence shows that SGLT-2 inhibitors administered to patients with CKD with or without T2DM reduce the risk of the primary cardiovascular outcome and the primary renal outcome. Likewise, moderate quality of evidence supports that SGLT-2 inhibitors decrease the hazard for all-cause mortality and CV death outcomes. However, we found low quality evidence for the use of SGLT-2 inhibitors to reduce the risk of MACE and HF hospitalizations.

The test for subgroup differences between patients with and without T2DM showed no significant subgroup effect for all the primary and secondary outcomes. This may suggest that the presence of T2DM does not modify the effect of SGLT-2 inhibitors in patients with CKD, so that any difference between patients with CKD with and without T2DM may be due to chance. However, there were insufficient studies reporting information for patients without T2DM. Out of six trials that included patients with CKD and with or without T2DM [10,11,32,38,56,57] , only the DAPA-CKD trial reported data for every outcome for the patients without T2DM [10,45,54] while EMPA-KIDNEY trial only reported such data for our primary renal outcome. Furthermore, we found high heterogeneity between studies in the subgroup of patients without T2DM for the primary renal outcome. In consequence, despite the apparent lack of subgroup differences in the analyses, we cannot reach a conclusive result.

Many systematic reviews have focused exclusively on patients with T2DM [12–15] with subgroup analyses for patients with CKD, while Tsai et al. [62] included only patients without T2DM. Our findings are largely consistent with these studies. There are other systematic reviews that included trials in patients both with and without T2DM [16,59,63,64]. Salah et al. [63] evaluated CV and renal outcomes in patients with cardiometabolic and kidney disease from the different clinical trials, regardless of the presence of CKD, T2DM and CV disease. However, their subgroup analysis for patients with CKD only comprised trials of patients with T2DM [35,36,39], thereby the patients with CKD without T2DM from trials such as the DAPA-CKD were not considered in the analysis. In contrast, Li et al. [59] focused on patients with CKD and included trials of patients with or without T2DM [10,32,38,57] and trials of diabetic patients [33–37,39,40]. However, while they calculated a subgroup estimate for diabetic patients, they did not conduct an analysis to test for subgroup differences. Chen et al. [64] only included trials in which the patients with CKD were the main population [10,33,40], and only evaluated a composite cardiorenal outcome. Finally, an update of a comprehensive systematic review [16] evaluated the effect of SGLT-2 inhibitors across all trials to date. However, in their analysis of patients with CKD, they only considered those patients who participated in CKD trials, such as the DAPA-CKD [10] or the EMPA-KIDNEY [11] trials. In contrast, in our analysis we included patients with CKD from every trial, increasing the number of total events per outcome.

Our main results on the primary CV outcome were similar to those reported in the available systematic reviews of studies on patients with or without T2DM [16,59], and studies on diabetic [14,15] and non-diabetic patients [62]. A recent updated meta-analysis of trials in patients without diabetes, also including CKD patients, showed that SGLT-2 inhibitors decreased the risk of CV death and HHF in non-diabetic patients [16]. This might suggest that the SGLT-2 inhibitors could have a protective effect in patients with CKD without T2DM. However, additional clinical trials are needed to consolidate the role of SGLT-2 inhibitors in this population. Our results show a narrower confidence interval than those previously described. Presumably, this difference is because more trials of SGLT-2 inhibitors in patients with CKD [10,11,33,40,52] have been published since the publication of the previous systematic reviews. Thus, we analyzed a greater number of patients with CKD, and this was reflected by a higher precision in our estimates.

Over time, the focus of clinical trials of SGLT-2 inhibitors efficacy has shifted from patients with T2DM and CV disease to patients with HF and CKD regardless of the presence of T2DM. In 2019, the CREDENCE trial [40] was the first to evaluate patients with CKD, though it only considered diabetic patients. Later, the DAPA-HF [38], EMPEROR [32,57] and DELIVER [56] trials assessed the effect of SGLT-2 inhibitors on patients with HF with or without T2DM. The first two trials that included patients with CKD with or without T2DM were DAPA-CKD [10] in 2020, and EMPA-KIDNEY [11] in 2022. A higher number of patients developed the primary CV outcome in the HF trials, which accounts for the higher weight these trials received in our meta-analyses. This may be explained by the fact that all the patients we selected from those trials had both HF and CKD, while only 10.9% of patients in the DAPA-CKD trial had HF and only 26.7% of patients in the EMPA-KIDNEY trial had established cardiovascular disease.

In our study, we found a significant reduction of the hazard for the primary renal outcome in patients with CKD as well as in the diabetic subgroup. Our result is consistent with other systematic reviews [13,16,41,63], which have described that SGLT-2 inhibitors confer a strong protective effect for a composite renal outcome in the diabetic population. These findings were expected, as these medications help control T2DM, which is a major risk factor for the development and progression of CKD [65]. On the other hand, we did not find a significant hazard reduction for the renal outcome in the subgroup of patients without T2DM. We attribute this to the small number of studies (n=2) included in this subgroup and the high heterogeneity between these two studies [10,11], which usually leads to a low precision of the estimated between‐study variance when using the random-effects model [66]. In contrast, another systematic review [16] analyzed the data from patients with CKD without T2DM from the same clinical trials [10,11] and found a significant protective effect. We believe their result is different to ours due to 2 reasons. First, they redefined the renal composite outcome to include a sustained ≥50% decline in eGFR, which decreased the total number of events; and second, they used different statistical methods of analysis. They also reported a significant decrease in the risk of kidney disease progression in diabetic nephropathy and other non-diabetic causes of CKD such as hypertension and glomerular diseases. This highlights that SGLT-2 inhibitors may improve renal function regardless of the glucose level control.

We found varied effects in the secondary outcomes in the literature compared to our analyses. For example, we obtained a significant result for the all-cause mortality outcome in patients with or without T2DM, in contrast to another systematic review in the same population [59]. Meanwhile, we found a significant protective effect for this outcome in the subgroup of CKD patients with T2DM, similarly to Arnott et al. [15]. However, another review of diabetic patients with CKD [13] did not reach the same finding. Our analysis coincides with a review [14] that did not find a protective effect for the MACE outcome in patients with CKD and T2DM, while other reviews [12,13,15] have shown otherwise. We believe that our analysis was affected by the inclusion of the results from Wada et al. [52], which was likely underpowered, as evidenced by the wide confidence intervals they reported. Regarding the cardiovascular death outcome, we found a significant protective effect in patients with CKD with or without T2DM, and in those with CKD and T2DM. Two systematic reviews [15,59] reported comparable results to the ones obtained from our analysis, but other systematic reviews did not [13,14,63]. Additionally, we found that SGLT-2 inhibitors have a strong protective effect for the hospitalization for HF outcome. Preceding systematic reviews also found a significant reduction of the hazard of hospitalization for HF with this treatment [13,15,59,63]. Overall, we believe that this variability on the results across systematic reviews is mostly explained by the gradual increase of published clinical trials in patients with CKD over time.

Independently of their original glucose-lowering effect, SGLT-2 inhibitors have other cardiorenal protective mechanisms beneficial for non-diabetic patients with CKD. Research on mice has shown that these medications increase natriuresis, which in turn decreases the systemic blood pressure and the intraglomerular pressure, and in consequence reduces glomerular hyperfiltration [6]. Additionally, anti-inflammatory and anti-fibrotic effects on tubular cells have also been described in rats [7,8]. According to a study on mice, all these effects may lead to a reduction of albuminuria; however, the exact mechanism is unclear [9]. Moreover, post hoc analyses of included trials suggest that SGLT-2 inhibitors reduce urinary markers of tubular damage such as KIM-1 [67] and remark that these drugs have beneficial effects in patients with HbA1c <7% [68]. Recently, it has been proposed that SGLT-2 inhibitors enhance cellular autophagy and housekeeping by stimulating nutrient deprivation signaling as well as inhibiting nutrient surplus signaling. This effect could explain the cardiorenal benefits of these drugs, and has even been evidenced in cells that do not express SGLT-2 proteins, suggesting that these drugs may act on other molecular targets such as sirtuins and mTOR

[69].

To the best of our knowledge, this is the first systematic review that evaluated patients with CKD according to the definition of each trial, which often considered the presence of albuminuria. This allowed us to include patients with CKD with a preserved eGFR but a high albuminuria. Previous systematic reviews [12–15,59,63] established an eGFR < 60 ml/min/1.73 m^2^ as the sole criterion for the diagnosis of CKD, potentially excluding data from patients with CKD that do not fulfill this criterion. Our approach may increase the external validity of our estimates. Furthermore, no other systematic review has assessed clinical CV and renal outcomes in patients with CKD with or without T2DM from every available trial to date, regardless of whether the trials only included patients with CKD or not. Finally, while there was some variability in the individual components of the composite kidney outcome in each trial, they mostly comprised clinically relevant markers such as doubling of serum creatinine, strong decline of eGFR, end-stage kidney disease, or renal death.

We recognize several limitations in our study. First, most of the clinical trials in our analysis [32,34,39,56,57] considered the patients with CKD as a subgroup rather than as their main study population. Thus, these studies were not originally designed to detect the treatment effect in these patients. Second, we summarized the effect of five SGLT-2 inhibitors (canagliflozin, dapagliflozin, empagliflozin, ertugliflozin and sotagliflozin) without analyzing them individually. Hence, the protective effects found in the CKD population may not be applicable to all the members of the gliflozin family. Third, some baseline and outcome data from the trials were missing. Consequently, we could not incorporate these elements into our results. Fourth, we could only extract outcome data of patients with CKD without T2DM from 2 trials, which severely limited the external validity of our subgroup analyses. Finally, we did not evaluate the effect of SGLT-2 inhibitors according to specific causes of non-diabetic CKD, as we considered that the number of trials that reported this information was very low (n=2).

To date, the role of empagliflozin and dapagliflozin is well established in patients with HF without diabetes [70]. While only dapagliflozin is currently indicated for patients with CKD without T2DM [71], the EMPA-KIDNEY trial has recently shown significant benefits of empagliflozin in the same population [11]. This could give rise to the approval of empagliflozin for the treatment of CKD in patients without diabetes. These benefits should be assessed in the rest of the gliflozin family. Additionally, we suggest that future trials use a standardized renal composite outcome to facilitate comparison between studies.

In conclusion, we found that in patients with CKD with or without T2DM, treatment with SGLT-2 inhibitors in addition to standard therapy conferred protection against CV and renal outcomes. Further research on patients with non-diabetic CKD should be done to confirm the utility of these medications in this specific population.

# **Acknowledgements**

# We thank Oscar J Ponce M.D. (O.J.P. - Knowledge and Evaluation Research Unit, Mayo Clinic, USA) for helping in the development of the research protocol, as well as in the study selection process and data analysis. We thank Paolo Wong M.D. (Universidad de Piura, Peru) for comments on the manuscript.References

1.	Vos T, Lim SS, Abbafati C, Abbas KM, Abbasi M, Abbasifard M, et al. Global burden of 369 diseases and injuries in 204 countries and territories, 1990–2019: a systematic analysis for the Global Burden of Disease Study 2019. Lancet. 2020;396: 1204–1222. doi: 10.1016/S0140-6736(20)30925-9

2.	Willis K, Cheung M, Slifer S. KDIGO 2012 Clinical Practice Guideline for the Evaluation and Management of Chronic Kidney Disease. Kidney International Supplements. 2013;3: 163. Available from: https://kdigo.org/guidelines/ckd-evaluation-and-management/

3.	National Institute for Health and Care Excellence. Chronic kidney disease: assessment and management (NG203). NICE. 2021; 77. Available from: https://www.nice.org.uk/guidance/ng203

4.	American Diabetes Association. Professional Practice Committee: Standards of Medical Care in Diabetes—2022. Diabetes Care. 2021;45: S3. doi: 10.2337/dc22-Sppc

5.	Cosentino F, Grant PJ, Aboyans V, Bailey CJ, Ceriello A, Delgado V, et al. 2019 ESC Guidelines on diabetes, pre-diabetes, and cardiovascular diseases developed in collaboration with the EASD: The Task Force for diabetes, pre-diabetes, and cardiovascular diseases of the European Society of Cardiology (ESC) and the European Association for the Study of Diabetes (EASD). Eur Heart J. 2020;41: 255–323. doi: 10.1093/eurheartj/ehz486

6.	Onishi A, Fu Y, Patel R, Darshi M, Crespo-Masip M, Huang W, et al. A role for tubular Na+/H+ exchanger NHE3 in the natriuretic effect of the SGLT2 inhibitor empagliflozin. Am J Physiol Renal Physiol. 2020;319: F712–F728. doi: 10.1152/ajprenal.00264.2020

7.	Ojima A, Matsui T, Nishino Y, Nakamura N, Yamagishi S. Empagliflozin, an Inhibitor of Sodium-Glucose Cotransporter 2 Exerts Anti-Inflammatory and Antifibrotic Effects on Experimental Diabetic Nephropathy Partly by Suppressing AGEs-Receptor Axis. Horm Metab Res. 2015;47: 686–692. doi: 10.1055/s-0034-1395609

8.	Castoldi G, Carletti R, Ippolito S, Colzani M, Barzaghi F, Stella A, et al. Renal Anti-Fibrotic Effect of Sodium Glucose Cotransporter 2 Inhibition in Angiotensin II-Dependent Hypertension. Am J Nephrol. 2020;51: 119–129. doi: 10.1159/000505144

9.	Gembardt F, Bartaun C, Jarzebska N, Mayoux E, Todorov VT, Hohenstein B, et al. The SGLT2 inhibitor empagliflozin ameliorates early features of diabetic nephropathy in BTBR ob/ob type 2 diabetic mice with and without hypertension. Am J Physiol Renal Physiol. 2014;307: F317–F325. doi: 10.1152/ajprenal.00145.2014

10.	Heerspink HJL, Stefánsson BV, Correa-Rotter R, Chertow GM, Greene T, Hou F-F, et al. Dapagliflozin in Patients with Chronic Kidney Disease. N Engl J Med. 2020;383: 1436–1446. doi: 10.1056/NEJMoa2024816

11.	The EMPA-KIDNEY Collaborative Group. Empagliflozin in Patients with Chronic Kidney Disease. N Engl J Med. 2022;388: 117-127. doi: 10.1056/NEJMoa2204233

12.	Zelniker TA, Wiviott SD, Raz I, Im K, Goodrich EL, Bonaca MP, et al. SGLT2 inhibitors for primary and secondary prevention of cardiovascular and renal outcomes in type 2 diabetes: a systematic review and meta-analysis of cardiovascular outcome trials. Lancet. 2019;393: 31–39. doi: 10.1016/S0140-6736(18)32590-X

13.	Toyama T, Neuen BL, Jun M, Ohkuma T, Neal B, Jardine MJ, et al. Effect of SGLT2 inhibitors on cardiovascular, renal and safety outcomes in patients with type 2 diabetes mellitus and chronic kidney disease: A systematic review and meta-analysis. Diabetes Obes Metab. 2019;21: 1237–1250. doi: 10.1111/dom.13648

14.	McGuire DK, Shih WJ, Cosentino F, Charbonnel B, Cherney DZI, Dagogo-Jack S, et al. Association of SGLT2 Inhibitors With Cardiovascular and Kidney Outcomes in Patients With Type 2 Diabetes. JAMA Cardiol. 2021;6: 1–11. doi: 10.1001/jamacardio.2020.4511

15.	Arnott C, Li Q, Kang A, Neuen BL, Bompoint S, Lam CSP, et al. Sodium‐Glucose Cotransporter 2 Inhibition for the Prevention of Cardiovascular Events in Patients With Type 2 Diabetes Mellitus: A Systematic Review and Meta‐Analysis. J Am Heart Assoc. 2020. doi: 10.1161/JAHA.119.014908

16.	Baigent C, Emberson JonathanR, Haynes R, Herrington WG, Judge P, Landray MJ, et al. Impact of diabetes on the effects of sodium glucose co-transporter-2 inhibitors on kidney outcomes: collaborative meta-analysis of large placebo-controlled trials. Lancet. 2022;400: 1788–1801. doi: 10.1016/S0140-6736(22)02074-8

17.	Page MJ, McKenzie JE, Bossuyt PM, Boutron I, Hoffmann TC, Mulrow CD, et al. The PRISMA 2020 statement: an updated guideline for reporting systematic reviews. BMJ. 2021;372: n71. doi: 10.1136/bmj.n71

18.	Sterne JAC, Savović J, Page MJ, Elbers RG, Blencowe NS, Boutron I, et al. RoB 2: a revised tool for assessing risk of bias in randomised trials. BMJ. 2019;366: l4898. doi: 10.1136/bmj.l4898

19.	McGuinness LA, Higgins JPT. Risk-of-bias VISualization (robvis): An R package and Shiny web app for visualizing risk-of-bias assessments. Res Synth Methods. n/a. doi: 10.1002/jrsm.1411

20.	Schünemann H H, Brożek J, Guyatt G, Oxman A. GRADE handbook for grading quality of evidence and strength of recommendations. The GRADE Working Group; 2013. Available from: guidelinedevelopment.org/handbook

21.	Schünemann H H, Brożek J, Guyatt G, Oxman A. Chapter 5: Quality of evidence. In: GRADE handbook for grading quality of evidence and strength of recommendations. The GRADE Working Group; 2013. Available from: guidelinedevelopment.org/handbook

22.	McMaster University and Evidence Prime. GRADEpro GDT: GRADEpro Guideline Development Tool. 2022. Available from: gradepro.org

23.	Paule RC, Mandel J. Consensus Values and Weighting Factors. J Res Natl Bur Stand. 1982;87: 377. doi: 10.6028/jres.087.022

24.	Hartung J, Knapp G. A refined method for the meta-analysis of controlled clinical trials with binary outcome. Stat Med. 2001;20: 3875–3889. doi: 10.1002/sim.1009

25.	Deeks JJ, Higgins JPT, Altman DG. Chapter 10: Analysing data and undertaking meta-analyses. In: Cochrane Handbook for Systematic Reviews of Interventions version 6.3 (updated February 2022). Cochrane; 2022. Available from: www.training.cochrane.org/handbook

26.	Borenstein M, Hedges LV, Higgins J, Rothstein HR. Introduction to meta-analysis. Second edition. Hoboken: Wiley; 2021.

27.	Altman DG. Statistics Notes: Interaction revisited: the difference between two estimates. BMJ. 2003;326: 219–219. doi: 10.1136/bmj.326.7382.219

28.	Egger M, Davey Smith G, Schneider M, Minder C. Bias in meta-analysis detected by a simple, graphical test. BMJ. 1997;315: 629–634. doi: 10.1136/bmj.315.7109.629

29.	R Core Team. R: A language and environment for statistical computing. Vienna, Austria: R Foundation for Statistical Computing; 2021. Available from: https://www.R-project.org/

30.	RStudio Team. RStudio: Integrated Development for R. Boston, MA: RStudio, PBC; 2022. Available from: http://www.rstudio.com/

31.	Schwarzer G, Carpenter JR, Rücker G. Meta-Analysis with R. Springer International Publishing; 2015.

32.	Packer M, Anker SD, Butler J, Filippatos G, Pocock SJ, Carson P, et al. Cardiovascular and Renal Outcomes with Empagliflozin in Heart Failure. N Engl J Med. 2020;383: 1413–1424. doi: 10.1056/NEJMoa2022190

33.	Bhatt DL, Szarek M, Pitt B, Cannon CP, Leiter LA, McGuire DK, et al. Sotagliflozin in Patients with Diabetes and Chronic Kidney Disease. N Engl J Med. 2021;384: 129–139. doi: 10.1056/NEJMoa2030186

34.	Bhatt DL, Szarek M, Steg PG, Cannon CP, Leiter LA, McGuire DK, et al. Sotagliflozin in Patients with Diabetes and Recent Worsening Heart Failure. N Engl J Med. 2021;384: 117–128. doi: 10.1056/NEJMoa2030183

35.	Wanner C, Inzucchi SE, Lachin JM, Fitchett D, von Eynatten M, Mattheus M, et al. Empagliflozin and Progression of Kidney Disease in Type 2 Diabetes. N Engl J Med. 2016;375: 323–334. doi: 10.1056/NEJMoa1515920

36.	Wiviott SD, Raz I, Bonaca MP, Mosenzon O, Kato ET, Cahn A, et al. Dapagliflozin and Cardiovascular Outcomes in Type 2 Diabetes. N Engl J Med. 2019;380: 347–357. doi: 10.1056/NEJMoa1812389

37.	Cannon CP, Pratley R, Dagogo-Jack S, Mancuso J, Huyck S, Masiukiewicz U, et al. Cardiovascular Outcomes with Ertugliflozin in Type 2 Diabetes. N Engl J Med. 2020;383: 1425–1435. doi: 10.1056/NEJMoa2004967

38.	McMurray JJV, Solomon SD, Inzucchi SE, Køber L, Kosiborod MN, Martinez FA, et al. Dapagliflozin in Patients with Heart Failure and Reduced Ejection Fraction. N Engl J Med. 2019;381: 1995–2008. doi: 10.1056/NEJMoa1911303

39.	Neal B, Perkovic V, Mahaffey KW, de Zeeuw D, Fulcher G, Erondu N, et al. Canagliflozin and Cardiovascular and Renal Events in Type 2 Diabetes. N Engl J Med. 2017;377: 644–657. doi: 10.1056/NEJMoa1611925

40.	Perkovic V, Jardine MJ, Neal B, Bompoint S, Heerspink HJL, Charytan DM, et al. Canagliflozin and Renal Outcomes in Type 2 Diabetes and Nephropathy. N Engl J Med. 2019;380: 2295–2306. doi: 10.1056/NEJMoa1811744

41.	Zelniker TA, Raz I, Mosenzon O, Dwyer JP, Heerspink HHJL, Cahn A, et al. Effect of Dapagliflozin on Cardiovascular Outcomes According to Baseline Kidney Function and Albuminuria Status in Patients With Type 2 Diabetes: A Prespecified Secondary Analysis of a Randomized Clinical Trial. JAMA Cardiol. 2021;6: 801–810. doi: 10.1001/jamacardio.2021.0660

42.	Cherney DZI, Charbonnel B, Cosentino F, Dagogo-Jack S, McGuire DK, Pratley R, et al. Effects of ertugliflozin on kidney composite outcomes, renal function and albuminuria in patients with type 2 diabetes mellitus: an analysis from the randomised VERTIS CV trial. Diabetologia. 2021;64: 1256–1267. doi: 10.1007/s00125-021-05407-5

43.	Zannad F, Ferreira JP, Pocock SJ, Zeller C, Anker SD, Butler J, et al. Cardiac and Kidney Benefits of Empagliflozin in Heart Failure Across the Spectrum of Kidney Function: Insights From EMPEROR-Reduced. Circulation. 2021;143: 310–321. doi: 10.1161/CIRCULATIONAHA.120.051685

44.	Jhund PS, Solomon SD, Docherty KF, Heerspink HJL, Anand IS, Böhm M, et al. Efficacy of Dapagliflozin on Renal Function and Outcomes in Patients With Heart Failure With Reduced Ejection Fraction. Circulation. 2021;143: 298–309. doi: 10.1161/CIRCULATIONAHA.120.050391

45.	Wheeler DC, Stefánsson BV, Jongs N, Chertow GM, Greene T, Hou FF, et al. Effects of dapagliflozin on major adverse kidney and cardiovascular events in patients with diabetic and non-diabetic chronic kidney disease: a prespecified analysis from the DAPA-CKD trial. Lancet Diabetes Endocrinol. 2021;9: 22–31. doi: 10.1016/S2213-8587(20)30369-7

46.	Jardine MJ, Zhou Z, Mahaffey KW, Oshima M, Agarwal R, Bakris G, et al. Renal, Cardiovascular, and Safety Outcomes of Canagliflozin by Baseline Kidney Function: A Secondary Analysis of the CREDENCE Randomized Trial. J Am Soc Nephrol. 2020;31: 1128–1139. doi: 10.1681/ASN.2019111168

47.	Neuen BL, Ohkuma T, Neal B, Matthews DR, de Zeeuw D, Mahaffey KW, et al. Relative and Absolute Risk Reductions in Cardiovascular and Kidney Outcomes With Canagliflozin Across KDIGO Risk Categories: Findings From the CANVAS Program. Am J Kidney Dis. 2021;77: 23-34.e1. doi: 10.1053/j.ajkd.2020.06.018

48.	Mosenzon O, Wiviott SD, Cahn A, Rozenberg A, Yanuv I, Goodrich EL, et al. Effects of dapagliflozin on development and progression of kidney disease in patients with type 2 diabetes: an analysis from the DECLARE-TIMI 58 randomised trial. Lancet Diabetes Endocrinol. 2019;7: 606–617. doi: 10.1016/S2213-8587(19)30180-9

49.	Neuen BL, Ohkuma T, Neal B, Matthews DR, de Zeeuw D, Mahaffey KW, et al. Cardiovascular and Renal Outcomes With Canagliflozin According to Baseline Kidney Function. Circulation. 2018;138: 1537–1550. doi: 10.1161/CIRCULATIONAHA.118.035901

50.	Wanner C, Lachin JM, Inzucchi SE, Fitchett D, Mattheus M, George J, et al. Empagliflozin and Clinical Outcomes in Patients With Type 2 Diabetes Mellitus, Established Cardiovascular Disease, and Chronic Kidney Disease. Circulation. 2018;137: 119–129. doi: 10.1161/CIRCULATIONAHA.117.028268

51.	Mc Causland FR, Claggett BL, Vaduganathan M, Desai AS, Jhund P, de Boer RA, et al. Dapagliflozin and Kidney Outcomes in Patients With Heart Failure With Mildly Reduced or Preserved Ejection Fraction: A Prespecified Analysis of the DELIVER Randomized Clinical Trial. JAMA Cardiol. 2023;8(1): 56-65. doi: 10.1001/jamacardio.2022.4210

52.	Wada T, Mori‐Anai K, Takahashi A, Matsui T, Inagaki M, Iida M, et al. Effect of canagliflozin on the decline of estimated glomerular filtration rate in chronic kidney disease patients with type 2 diabetes mellitus: A multicenter, randomized, double‐blind, placebo‐controlled, parallel‐group, phase III study in Japan. J Diabetes Investig. 2022;13: 1981–1989. doi: 10.1111/jdi.13888

53.	Heerspink HJL, Sjöström CD, Jongs N, Chertow GM, Kosiborod M, Hou FF, et al. Effects of dapagliflozin on mortality in patients with chronic kidney disease: a pre-specified analysis from the DAPA-CKD randomized controlled trial. Eur Heart J. 2021;42: 1216–1227. doi: 10.1093/eurheartj/ehab094

54.	Persson F, Rossing P, Vart P, Chertow GM, Hou FF, Jongs N, et al. Efficacy and Safety of Dapagliflozin by Baseline Glycemic Status: A Prespecified Analysis From the DAPA-CKD Trial. Diabetes Care. 2021;44: 1894–1897. doi: 10.2337/dc21-0300

55.	Levin A, Perkovic V, Wheeler DC, Hantel S, George JT, von Eynatten M, et al. Empagliflozin and Cardiovascular and Kidney Outcomes across KDIGO Risk Categories: Post Hoc Analysis of a Randomized, Double-Blind, Placebo-Controlled, Multinational Trial. Clin J Am Soc Nephrol. 2020;15: 1433–1444. doi: 10.2215/CJN.14901219

56.	Solomon SD, McMurray JJV, Claggett B, de Boer RA, DeMets D, Hernandez AF, et al. Dapagliflozin in Heart Failure with Mildly Reduced or Preserved Ejection Fraction. N Engl J Med. 2022;387: 1089–1098. doi: 10.1056/NEJMoa2206286

57.	Anker SD, Butler J, Filippatos G, Ferreira JP, Bocchi E, Böhm M, et al. Empagliflozin in Heart Failure with a Preserved Ejection Fraction. N Engl J Med. 2021;385: 1451–1461. doi: 10.1056/NEJMoa2107038

58.	Zannad F, Kraus BJ, Zeller C, Pocock S, Packer M, Filippatos G, et al. EMPEROR-Preserved: Empagliflozin and Outcomes in Heart Failure with a Preserved Ejection Fraction and CKD. Hypertension and CVD. Live-Stream, Virtual only: American Society of Nephrology; 2021. Available from: https://www.asn-online.org/education/kidneyweek/2021/program-abstract.aspx?controlId=3639415

59.	Li N, Zhou G, Zheng Y, Lv D, Zhu X, Wei P, et al. Effects of SGLT2 inhibitors on cardiovascular outcomes in patients with stage 3/4 CKD: A meta-analysis. PLoS One. 2022;17: e0261986. doi: 10.1371/journal.pone.0261986

60.	Rådholm K, Figtree G, Perkovic V, Solomon SD, Mahaffey KW, de Zeeuw D, et al. Canagliflozin and Heart Failure in Type 2 Diabetes Mellitus. Circulation. 2018;138: 458–468. doi: 10.1161/CIRCULATIONAHA.118.034222

61.	Cherney DZI, McGuire DK, Charbonnel B, Cosentino F, Pratley R, Dagogo-Jack S, et al. Gradient of Risk and Associations With Cardiovascular Efficacy of Ertugliflozin by Measures of Kidney Function. Circulation. 2021;143: 602–605. doi: 10.1161/CIRCULATIONAHA.120.051901

62.	Tsai W-C, Hsu S-P, Chiu Y-L, Yang J-Y, Pai M-F, Ko M-J, et al. Cardiovascular and renal efficacy and safety of sodium-glucose cotransporter-2 inhibitors in patients without diabetes: a systematic review and meta-analysis of randomised placebo-controlled trials. BMJ Open. 2022;12: e060655. doi: 10.1136/bmjopen-2021-060655

63.	Salah HM, Al’Aref SJ, Khan MS, Al-Hawwas M, Vallurupalli S, Mehta JL, et al. Effect of sodium-glucose cotransporter 2 inhibitors on cardiovascular and kidney outcomes-Systematic review and meta-analysis of randomized placebo-controlled trials. Am Heart J. 2021;232: 10–22. doi: 10.1016/j.ahj.2020.10.064

64.	Chen H-B, Yang Y-L, Yu T-H, Li Y-H. SGLT2 inhibitors for the composite of cardiorenal outcome in patients with chronic kidney disease: A systematic review and meta-analysis of randomized controlled trials. Eur J Pharmacol. 2022;936: 175354. doi: 10.1016/j.ejphar.2022.175354

65.	Bikbov B, Purcell CA, Levey AS, Smith M, Abdoli A, Abebe M, et al. Global, regional, and national burden of chronic kidney disease, 1990–2017: a systematic analysis for the Global Burden of Disease Study 2017. Lancet. 2020;395: 709–733. doi: 10.1016/S0140-6736(20)30045-3

66.	Jackson D, Law M, Rücker G, Schwarzer G. The Hartung‐Knapp modification for random‐effects meta‐analysis: A useful refinement but are there any residual concerns? Stat Med. 2017;36: 3923–3934. doi: 10.1002/sim.7411

67.	Sen T, Koshino A, Neal B, Bijlsma MJ, Arnott C, Li J, et al. Mechanisms of action of the sodium‐glucose cotransporter‐2 (SGLT2) inhibitor canagliflozin on tubular inflammation and damage: A post hoc mediation analysis of the CANVAS trial. Diabetes Obes Metab. 2022;24: 1950–1956. doi: 10.1111/dom.14779

68.	Cahn A, Wiviott SD, Mosenzon O, Goodrich EL, Murphy SA, Yanuv I, et al. Association of Baseline HbA1c With Cardiovascular and Renal Outcomes: Analyses From DECLARE-TIMI 58. Diabetes Care. 2022;45: 938–946. doi: 10.2337/dc21-1744

69.	Packer M. Critical Reanalysis of the Mechanisms Underlying the Cardiorenal Benefits of SGLT2 Inhibitors and Reaffirmation of the Nutrient Deprivation Signaling/Autophagy Hypothesis. Circulation. 2022;146: 1383–1405. doi: 10.1161/CIRCULATIONAHA.122.061732

70.	Heidenreich PA, Bozkurt B, Aguilar D, Allen LA, Byun JJ, Colvin MM, et al. 2022 AHA/ACC/HFSA Guideline for the Management of Heart Failure: A Report of the American College of Cardiology/American Heart Association Joint Committee on Clinical Practice Guidelines. Circulation. 2022;145: e895–e1032. doi: 10.1161/CIR.0000000000001063

71.	UK Kidney Association. Clinical Practice Guideline: Sodium-Glucose Co-transporter-2 (SGLT-2) Inhibition in Adults with Kidney Disease. 2021. Available from: https://ukkidney.org/sites/renal.org/files/UKKA%20guideline_SGLT2i%20in%20adults%20with%20kidney%20disease%20v1%2020.10.21.pdf

# Supporting information

**S1 Table. Definition of chronic kidney disease and the renal and cardiovascular composite outcomes in each study.** *We used an eGFR < 60 ml/min/1.73 m^2^ as the definition of chronic kidney disease. CKD, chronic kidney disease; CV, cardiovascular; eGFR, estimated glomerular filtration rate; HF, heart failure; NA, not available; UACR, urine albumin-to-creatinine ratio.

**S2 Table. Search Strategy.**

**S3 Table. Clinical trial baseline characteristics ordered according to publication date.** Data calculated from other publications of the included trial is shaded blue, since it was not directly available in the published articles. ACEI, angiotensin-converting enzyme inhibitors; ARB, angiotensin receptor blockers; CKD, chronic kidney disease; CV, cardiovascular; DPP-4, dipeptidyl peptidase-4; eGFR, estimated glomerular filtration rate; GLP-1, glucose-like peptide-1; HF, Heart failure; IQR, interquartile range; MRA, mineralocorticoid receptor antagonist; n, absolute frequency; NA, not available; SD, standard deviation; SGLT-2, sodium-glucose cotransporter 2; T2DM, type 2 diabetes mellitus; UACR, urine albumin-to-creatinine ratio.

**S4 Table. Summary of the evidence.**

**S1 Fig. Risk of bias assessment.** (A) Traffic light plot. (B) Bar plot.

**S2 Fig. Sensitivity analysis: Effects of SGLT-2 inhibitors on the primary outcomes in patients with CKD and an eGFR < 60 ml/min/1.73 m^2^**. (A) Primary cardiovascular outcome. (B) Primary renal outcome. ^¶^Synthetic estimate created with a fixed effects model meta-analysis. CI, confidence interval; CKD, chronic kidney disease; HR, hazard ratio; NA, not available.

**S3 Fig. Sensitivity analysis: Effects of SGLT-2 inhibitors on the secondary outcomes in patients with CKD and an eGFR < 60 ml/min/1.73 m^2^**. (A) All-cause mortality. (B) MACE outcome. (C) CV death. (D) Hospitalization for HF. ^¶^Synthetic estimate created with a fixed effects model meta-analysis. CI, confidence interval; CKD, chronic kidney disease; HR, hazard ratio; NA, not available.

**S4 Fig. Reporting bias assessment: Funnel plots and Egger’s tests.** (A) Primary cardiovascular outcome. (B) Primary renal outcome. (C) All-cause mortality. (D) MACE outcome. (E) CV death. (F) Hospitalization for HF.

**S1 Database.**

**S1 Checklist.**
