# Supplementary material for: The effect of sodium-glucose cotransporter 2 inhibitors in patients with chronic kidney disease with or without type 2 diabetes mellitus on cardiovascular and renal outcomes: A systematic review and meta-analysis
Source: PLoS One. 2023 Nov 29;18(11):e0295059. doi: 10.1371/journal.pone.0295059 (PMC10686459; doi:10.1371/journal.pone.0295059)
Supplement: Attachment — Submitted filename: Response to reviewers.docx [file pone.0295059.s019.docx]

**Dear reviewers,**

Thank you for your further suggestions about our manuscript. We have implemented the changes and improved our manuscript accordingly.

**Sincerely,**

**Marcelo Reategui-Diaz**

**Reviewer #1:**

[1] The Discussion section appears quite lengthy. I recommend that the authors work on making it more concise.

**Response**: We agree with your suggestion, and thus we have reduced the number of words from most of the Discussion section paragraphs. With these changes, the word count was reduced from 2236 to 1623 words.

[2] When explaining the subgroup analysis based on the type of SGLT-2 inhibitors, please consistently use the term "the type of SGLT-2 inhibitors" instead of simply referring to "the SGLT-2 inhibitor" throughout the manuscript.

**Response**: We agree with your suggestion. In the new version, we have used the term "the type of SGLT-2 inhibitors" more consistently across the manuscript.

[3] In order to emphasize the significance and relevance of the current study, please indicate that this study aimed to include all the available patients with CKD from the current literature, not only those from clinical trials dedicated to CKD, but also those where CKD patients were included as a subgroup.

**Response**: We agree with your suggestion. We modified the Abstract, Introduction, Methods and Discussion sections to emphasize that our study aimed to assess the impact of SGLT-2 inhibitors on the entire CKD population in the current literature, including both CKD trials and trials in which CKD patients were a subgroup.

[4] In the third paragraph of the Introduction section (lines 57 – 61), I would like to recommend the authors to rephrase the sentences. Because the aim of the present study appears in the next paragraph, the sentence above should highlight the needs for an additional meta-analysis, while avoiding the term “our results”. For example, “However, to date, there has been no comprehensive systematic review assessing the impact of SGLT-2 inhibitors on the entire CKD population, as provided in the available literature. Incorporating data from all CKD patients within clinical trials, not solely those designed exclusively for CKD patients but also those where CKD patients are included as a subgroup, could enhance the accuracy of effect estimates and enhance the applicability of the findings.” Please note that this is just a suggestion, and I hope the authors can refine the sentences further.

**Response**: We modified the Introduction section to incorporate your suggestions in this paragraph (lines 57-62).

[5] Likewise, in the last paragraph of the Introduction section (lines 62 – 64), consider rephrasing it as follows: "This systematic review aims to assess the cardiovascular and renal effects of SGLT-2 inhibitors in all available CKD patients in the current literature and to evaluate whether the presence of T2DM modifies their overall impact." Please note that this is just a suggestion.

**Response**: We agree with your suggestion. We modified the Introduction section to incorporate your suggestion in this paragraph (lines 63-65).

[6] In the Discussion section (line 299), please specify in the sentence that the total number of participants with CKD was compared to the study by Baigent et al.

**Response**: We agree with your suggestion. We modified the Discussion section to incorporate your suggestion in this paragraph (line 286).

**Reviewer #2:**

The authors responded to all of the review questions one on one and included detailed evidence. In particular, authors assessed heterogeneity between CKD patients with and without T2DM and performed statistical tests for each outcome to confirm that there were no statistical differences between subgroups

**Response**: Thank you for your thorough evaluation of our manuscript.
