# Supplementary material for: The effect of sodium-glucose cotransporter 2 inhibitors in patients with chronic kidney disease with or without type 2 diabetes mellitus on cardiovascular and renal outcomes: A systematic review and meta-analysis
Source: PLoS One. 2023 Nov 29;18(11):e0295059. doi: 10.1371/journal.pone.0295059 (PMC10686459; doi:10.1371/journal.pone.0295059)
Supplement: Attachment — Submitted filename: Response to reviewers.docx [file pone.0295059.s020.docx]

**Dear reviewer,**

Thank you for your comments about the Discussion section of our manuscript. We have implemented the changes and improved our manuscript accordingly.

**Sincerely,**

**Marcelo Reategui-Diaz**

**Reviewer:**

I want to express my gratitude for the authors' dedication and time spent on the revision. The authors have made substantial efforts in addressing the comments from the previous revision. While I acknowledge the significant improvements in the manuscript, I still believe that the Discussion section could benefit from further refinement. Some paragraphs appear to disrupt the overall flow and readability. Specifically, the paragraph beginning with "Independently of their glucose-lowering effect..." seems inconsistent with the surrounding paragraphs. Additionally, the paragraph that starts with "To date, the role of empagliflozin and dapagliflozin..." doesn't seem to flow seamlessly with the adjacent paragraphs.
Would it be possible to consider reorganizing the paragraphs (not only the above two paragraphs I have mentioned, but also other paragraphs) within the Discussion section?

**Response**: We agree with your comments. In consequence, we have sorted the paragraphs to follow a more logical order. We also paid particular attention to the two paragraphs that were highlighted in your comment. We have merged those paragraphs with adjacent paragraphs seamlessly, so that the readability and connection of ideas are improved.
